# Electronic Structure and Electron Delocalization in Bare and Dressed Boron Pentamer Clusters

**DOI:** 10.1021/acs.jpca.1c02305

**Published:** 2021-06-11

**Authors:** Jose M. Mercero, Jesus M. Ugalde

**Affiliations:** Kimika Fakultatea, Euskal Herriko Unbertsitatea (UPV/EHU), Donostia International Physics Center (DIPC), P.K. 1072, 20080 Donostia, Euskadi, Spain

## Abstract

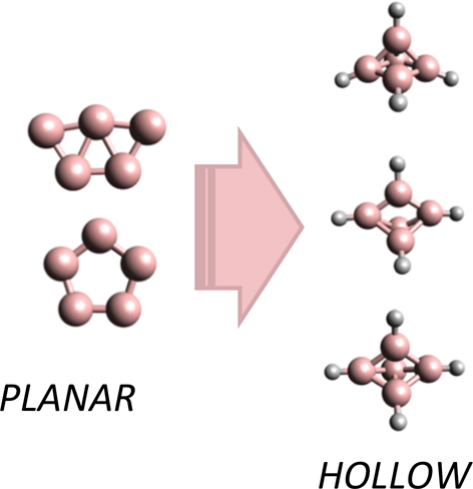

The electronic structures of the lowest energy spin-states of the cationic, neutral and anionic bare boron pentamer clusters have been investigated by means of high level multiconfigurational type calculations, in view of the large static and dynamical electron correlation effects for these species. We found that  resembles a singlet spin-state perfect pentagon, which bears no intra-annular chemical bonding interactions, as shown by our analysis of the electron delocalization carried out in terms of the normalized Giambiagi ring-current index, and the total and adjacent atom-pair delocalization indices. However, its lowest-energy triplet and quintet spin-state isomers have *C*_2*v*_ symmetry, with large intra-annular chemical bonding interactions. This geometrical feature extends to both the neutral and the anionic species. Namely, the lowest-energy isomers of boron pentamer neutral and anionic clusters have peripheral and intra-annular sizable bonding interactions reflected in the delocalization of both π- and σ-type valence natural orbitals over the whole molecular plane, which impart large structural stability. In accordance to our calculations, the lowest energy triplet spin-state isomer of the anionic boron pentamer cluster has *C*_2_ symmetry, and consequently, it should show optical activity. Finally, we have studied the change of the geometrical structure of the boron pentamer clusters from planar to compact three-dimensional structures caused by the bonding of ligands to the boron atoms. Our explicit all-electron calculations have been rationalized in terms of the shell-closure of the delocalized valence orbitals of the clusters as predicted by the jellium model extended to nonspherical confinement potentials, circumscribing the role of the ligand to modulate the total number of valence electrons assigned to the core cluster.

## Introduction

Boron clusters have been extensively explored since Hanley et al.^1^ reported their seminal work on the mass abundance distribution as a function of the number of atoms, in the range *n* = 1–20, of boron clusters generated by laser ablation. Later, La Placa et al.^2^ extended the clusters’ sizes to *n* = 52 and provided ample experimental data for theoreticians to look at their geometrical structures, as this information is absent from these experiments. This permitted researchers to find out and rationalize their properties in terms of their corresponding electronic structures.^3,4^

In the course of this research, it was soon realized that boron poses a number of thrilling challenges to theory, as bonding between boron atoms is expected to be more complex than that of its periodic-table adjacent elements, and consequently distinct geometrical features must be expected to result in boron clusters.

Thus, boron can form both two- and three-center bonds that can ultimately yield a vast plethora of geometrical structures unique to boron clusters in both two-,^5^ and three-dimensional^6^ arrangements. In this vein, it is worth noticing that the interplay of these bonding modes of boron yields as many of 16 bulk allotropes based on icosahedral B_12_ units, along with a number of other small interstitial clusters and fused supericosahedra.^7^

However, boron clusters of less than 15 atoms are known to possess pseudoplanar minimum energy isomers, with substantial electron delocalization which resemble aromaticity features of carbon-like molecules.^8^ This was shown by Fowler and Ugalde^9^ who put forward that the large electron delocalization found for the minimum energy isomer of the  cluster stems from a valence molecular orbital structure similar to that of benzene; namely, the valence molecular orbitals are arranged in increasing energy as one molecular orbital with no nodes in the molecular plane, followed by two pseudodegenerate molecular orbitals of π-symmetry with one node each. These are the occupied molecular orbitals. Then, the virtual molecular orbitals come as two pseudodegenerate molecular orbitals of π-symmetry with two nodes each, and finally one three-node molecular orbital. All the molecular orbitals are fully delocalized over the whole cluster. This interpretation was reinforced by considering the parent  cluster anion. Now, for the singlet spin state, the two-node pseudodegenerate molecular orbitals will be unequally populated, and consequently will trigger a Jahn–Teller distortion toward an oval-like shaped molecular geometry. This distortion was precisely documented by Fowler and Ugalde,^9^ thus lending support to its benzene-like electron delocalization to the point that  is best seen as a 6π aromatic molecule. More extensive studies on the aromaticity of planar boron cluster followed suit.^8^

The  bare cluster is interesting for it is one of two specially stable clusters found by Hanley et al.^1^ in their collision-induced dissociation (CID) experiments, carried out on the “magic number” clusters generated by laser ablation. The other specially stable cluster was .

The boron pentamer cluster has also been studied earlier. Thus, Morokuma et al.,^3^ assigned a pseudoplanar *C*_2_ symmetry structure for the most stable isomer of B_5_ (^2^B electronic state), and a planar *C*_2*v*_ for the  (^1^A_1_ state) cation, based on their single-reference MP4/6-31G(d) calculations. Later, Dixon et al.^10^ optimized the structures of the  (*n* = 5–13) clusters with the approximate B3LYP/6-31G(d) density functional and refined their energies with coupled-cluster theory CCSD(T) calculations with the aug-cc-pVηZ (η = D, T, and Q) basis sets extrapolated to the complete basis set limit (CBS) for the smaller clusters *n* = 5–9, and corrections from G3B3 calculations for the larger ones *n* = 10–13.

However, an in-depth consideration of the electronic structure of the boron pentamer cluster suggests that static electron correlation effects might play a role, since electron deficient clusters, like B_5_, often have partially filled pseudodegenerate valence molecular orbitals.^11^ For instance,  possesses four valence electrons to be accommodated in four pseudodegenerate valence molecular orbitals.^12^ In this respect, it is worth mentioning that recent high level multireference electronic structure calculations have revealed the large multiconfigurational character of the electronic structure of the boron pentamer, making mandatory the explicit consideration of the static electron correlation effects, which are absent in single-reference schemes. In particular, multiconfigurational self-consistent field complemented with multiconfigurational quasi-degenerate perturbation theory calculations predict that the most stable isomer of B_5_ is a distorted pentagon with *C*_2*v*_ symmetry (^2^B_2_ state),^13^ and that of the  cation is a perfect pentagon with *D*_5*h*_ symmetry (^1^A_1_′ state).^14^ For the  cluster anion’s most stable isomer geometry, the earlier multiconfigurational self-consistent field calculations of Boldyrev et al.^15^ have never been complemented by multiconfigurational quasi-degenerate perturbation theory calculations for accurate total energy determination. Nonetheless, it is worth noticing that the *C*_2*v*_ symmetry (^1^A_1_ state) structure proposed by Wang, Boldyrev, et al.,^8^ based on their PBE0/6-311+G* geometry optimizations supplemented with CCSD(T) single point calculation for the refinement of the energies, nicely matches the experimental vertical detachment energies (VDEs) of the boron pentamer cluster anion.

Bare clusters are prone to collapsing into larger units when put in close proximity of each other.^16^ Stabilization of clusters toward coalescence is provided by passivating them with an outer shell of ligands. This provides chemical stability to the cluster, but often changes dramatically their chemical and physical properties.^17^ Indeed, it has been confirmed that the effect of the outer shell ligands must be taken into consideration for understanding the electronic properties of ligand stabilized clusters.^18^

Passivation of boron clusters has a long history in chemistry, which goes back to the seminal work of Lipscomb,^19^ and boron clusters have been found to yield a formidable richness of chemistry.^20,21^ The hydrides of boron clusters with generic formula (BH)_*n*_, known as boranes, expand into a wide range of compounds, some of which are very reactive, in particular toward electron donors given their electron deficient nature. Some of them are even pyrophoric upon air exposure or react violently with water. Others, however, are remarkably stable. Thus, the icosahedral dodecaborate dianion, , is thermodynamically far more stable than benzene.^22^

In this vein, the recent synthesis of two dodecaborate radical anions, , containing a dense outer shells of (−OR) ligands that have been found to enhance the ^19^F nuclear magnetic resonance signals due to the transfer of spin polarization from the cluster’s electron radical to the ^19^F nuclei in dissolution dynamic nuclear polarization experiments (D-DNP),^23^ puts forward the enormous practical interest of the controlled passivation and functionalization of self-assembled ligand-stabilized clusters in current materials-design nanoscience.^24^

In this paper, we will analyze the electronic structures of the most stable and a few low-lying isomers of the various spin states of the boron pentamer cluster and its cationic and anionic derivatives, with the aim of understanding better their bonding patterns, geometries, and chemical properties. Furthermore, we will also study the passivation of the neutral cluster to unveil the physical basis of the change of the geometrical structure upon bonding of the outer ligand shell.

## Computational Details

The wave functions of electron deficient compounds have usually large multiconfigurational character due to the accessibility of several near-degenerated molecular orbitals for electrons to occupy. This is turn results in several configurations to be equally valid reference Slater determinants for the basic description of the electronic structure. Multiconfigurational methods constitute the natural approach to deal with such cases. Here, we have opted for a multiconfigurational self-consistent field (MCSCF) level of theory^25^ with four, five, and six active electrons for the cation, neutral moiety, and anion, respectively, in 15 orbitals using the augmented correlation-consistent polarized valence triple-ζ basis set,^26^ hereafter denoted as MCSCF(N,15)/aug-cc-PVTZ.

The reasoning for the selection of our Complete Active Space (CAS) for the calculation of the reference MCSCF wave function is based on Dixon’s analysis of the valence molecular orbitals of annular ring-like molecules made of main-group electron-deficient atoms.^10^ Dixon has established that p-type atomic orbitals do conform the active space which carries static electron correlation, and the s-type atomic orbitals mainly contribute dynamic electron correlation. Thus, for boron pentamers we have a 5 × 3 p-type atomic orbital which generates our 15 molecular orbitals of the CAS. For the neutral boron pentamer cluster we have 5 electrons in these orbitals, since each boron atom contributes one p-type electron. Cationic and anionic pentamer clusters follow suit (see Supporting Information for further details concerning the selection of the active space).

We have optimized the geometries and evaluated the harmonic vibrational frequencies of the lowest-lying isomers of the singlet, triplet, and quintet spin states of  charged clusters and of the doublet and quartet spin states of B_5_. Subsequently, multiconfigurational quasi-degenerate perturbation^27^ (MCQDPT) calculations were carried out on the MCSCF(N,15)/aug-cc-PVTZ optimized geometries. All core, valence, and virtual orbitals have been correlated in the MCQDPT calculations. This brings in the missed dynamical electron correlation in the reference MCSCF wave function. Inconsistencies caused by the so-called intruder states, which appear when the perturbation expansion of the reference MCSCF wave function has vanishingly small energy denominators, were remedied by shifting them by 0.02 au, as recommended earlier.^28^

Notice that we provided energies at the MCQDPT level of theory and not at the MCSCF level, which is used exclusively to set a reference wave function for the MCDQPT calculations.

All these calculations were carried out with the GAMESS program.^29^

## Results and Discussion

The cationic, neutral, and anionic boron pentamer clusters have already been studied by Boldyrev et al.,^30^ who also have reviewed earlier calculations. Notably, their calculations accounted satisfactorily for the most of salient features of the photoelectron detachment experiments of the  cluster anion. They carried electronic structure calculations at three levels of theory, namely, density functional theory (B3LYP), Møller–Plesset second-order perturbation theory (MP2) and coupled-cluster with single and double excitations and Davidson’s correction for triplet excitations (CCSD(T)). They used Pople’s 6-311+G* and Dunning’s aug-cc-pVXZ (X= D, T, Q) basis functions sets. Additionally, for the doublet spin state of B_5_, and the singlet spin state of the , they carried out multiconfigurational type calculations, i.e., CASSCF(7,8) and CASSCF(8,8), respectively.

However, careful inspection of the molecular orbitals of the lowest energy  is very suggestive that substantial static electron correlation effects may arise due the fractional occupation of the valence natural molecular orbitals of these species. Such an effect is best accounted for by means of multiconfigurational procedures, which generate the correct reference wave function. Then, subsequent perturbation calculations must be used to recover (most of) the missing dynamical electron correlation to yield an accurate and balanced description of the electron correlation effects.^31^

Thus, herein we will calculate the electronic structures of the lowest energy wave functions of the various spin states of all isomers at the same high-level static electron-correlation including multiconfigurational level of theory, complemented with multidegenerate perturbation theory type calculations to refine energies. Then, we will analyze the electron delocalization features of all these states in view of three indices, specifically designed for such purpose (vide infra), obtained from the optimized multiconfigurational wave functions. Furthermore, we will expand our study to consider the change of the geometrical structure of boron pentamer clusters upon ligation of protective substituents.

### Bare Boron Pentamer Clusters

The results of an extensive search of the singlet, triplet and quintet spin state potential energy surfaces at the MCSCF(N,15)/aug-cc-pTZVP level of theory, subsequently supplemented with single-point MCQDPT calculations, including all core, valence and virtual molecular orbitals in the perturbational space, shows that the most stable isomer of  corresponds to the *D*_5*h*_ symmetry ^1^A_1_′ singlet state. For the triplet spin a ^3^B_2_ state of *C*_2*v*_ symmetry bears the minimum energy, while for the quintet spin the *C*_2*v*_ symmetry ^5^A_1_ state is found to be the minimum energy isomer. Likewise calculations for the doublet and quartet spin state potential energy surfaces, show that the most stable isomer of the neutral B_5_ (*N* = 5) cluster has *C*_2*v*_ symmetry and ^2^B_2_ electronic state, while on the quartet spin surface, the lowest lying one corresponds to the ^4^A′ electronic state of the *C*_*s*_ symmetry planar isomer. Finally, the most stable isomer of the anionic cluster  corresponds to the *C*_2*v*_ symmetry ^1^A_1_ singlet spin electronic state, while for the triplet state we have found a *C*_2_ symmetry isomer with the ^3^B electronic structure, and for the quintet state, the lowest lying isomer is the *C*_2*v*_ symmetry isomer with ^5^A_1_ electronic structure.

**Figure 1:**
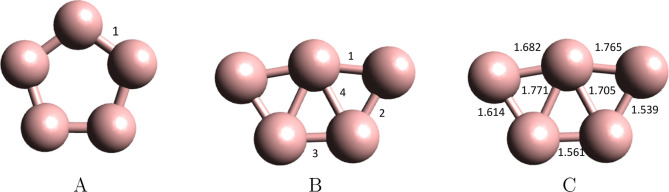
Sketch of the MCSCF(N,15)/aug-cc-pTZVP optimized molecular geometries, *D*_5*h*_ (A) and *C*_2*v*_/*C*_2_ (B), and bonding patterns of the isomers of the  (C) clusters. The MCSCF(N,15)/aug-cc-pTZVP optimum bond lengths for each of the electronic states of the isomers can be found in Table 1. The bond lengths of the B_5_ cluster’s *C*_*s*_ lowest-lying quartet spin-state, ^4^A′ isomer are shown in the right panel.

The obtained optimized geometries are sketched in Figure 1; the optimized bond lengths can be found in Table 1 and the relative energies in Table 2. Finally the calculated Cartesian coordinates, harmonic vibrational frequencies and zero-point harmonic energies for all structures discussed in the present investigation are listed in the Supporting Information.

**Table 1:** MCSCF(N,15)/aug-cc-pTZVP Optimum Bond Lengths of the  Clusters^a^

state	*R*(1)	*R*(2)	*R*(3)	*R*(4)
B_5_^+^				
^1^A_1_′(*D*_5*h*_)	1.550			
^3^B_2_(*C*_2*v*_)	1.618	1.577	1.593	2.025
^5^A_1_(*C*_2*v*_)	1.972	1.595	1.494	1.662
B_5_				
^2^B_2_(*C*_2*v*_)	1.590	1.567	1.546	1.871
^4^A′(*C*_*s*_)	see Figure 1C			
B_5_^–^				
^1^A_1_(*C*_2*v*_)	1.624	1.583	1.556	1.749
^3^B(*C*_2_)	1.615	1.575	1.555	1.816
^5^A_1_(*C*_2*v*_)	1.756	1.505	1.619	1.679

a See Figure 1, parts A, B. and D, for the meaning of the symbols.

**Table 2:** Lowest Lying MCQDPT/MCSCF(N,15)/aug-cc-pVTZ Singlet, Triplet, and Quintet Electronic States of the  Cluster Cation and Anion and Doublet and Quartet Electronic States of the B_5_ Neutral Cluster^a^

B_5_^+^		B_5_		B_5_^–^	
state	Δ*E*	state	Δ*E*	state	Δ*E*
^1^A_1_′(*D*_5*h*_)	0.00	^2^B_2_(*C*_2*v*_)	0.00	^1^A_1_(*C*_2*v*_)	0.00
^3^B_2_(*C*_2*v*_)	21.49	^4^A′(*C*_*s*_)	22.08	^3^B(*C*_2_)	9.07
^5^A_1_(*C*_2*v*_)	37.17			^5^A_1_(*C*_2*v*_)	29.03

a Δ*E* stands for the relative energy, in kcal/mol, including the zero-point vibrational energy corrections with respect to the lowest lying isomer of each charge state, namely, the  state for the cation, whose energy is −123.354588 hartree, the ^2^B_2_ state for the neutral, whose energy is −123.684798 hartree, and the ^1^A_1_ state for the anion, whose energy is −123.780549 hartree.

**Figure 2:**
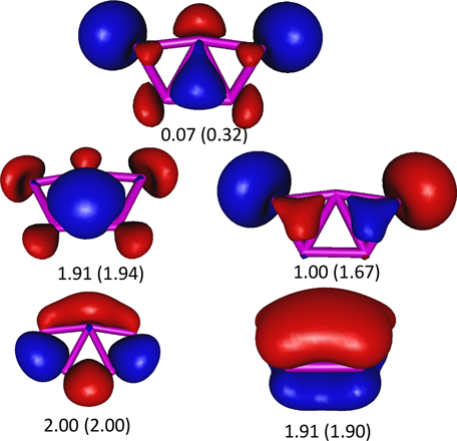
Isosurface representation of the delocalized bonding natural orbitals along with their occupation numbers, of the most stable isomer of the B_5_ cluster. The corresponding orbitals for  are indistiguishable in this representation, but their occupation numbers (shown in parentheses) differ slightly.

Our calculations predict an adiabatic electron detachment energy of 2.60 eV for the ^1^A_1_(*C*_2*v*_) ground state of the most stable isomer of the anionic , which is to be compared with the experimentally measured value of 2.33 ± 0.02 eV.^15^ Boldyrev et al.,^15^ based on the inspection of the B3LYP/6-311+G* Kohn–Sham molecular orbitals, ascribed the stability of the *C*_2*v*_ symmetry planar structures of the most stable isomer of  to two delocalized molecular orbitals, namely, the peripheral 4a_1_, and the π-type 1b_1_ molecular orbitals delocalized over the five boron atoms. The equivalent MCSCF(6,15)/aug-cc-pVTZ natural orbitals are shown in Figure 2 with occupation numbers 2.00 and 1.90, respectively. However, inspection of the high level MCSCF(6,15)/aug-cc-pVTZ molecular orbitals shows that the in addition to the molecular orbitals alluded to above, the radially delocalized a_1_ symmetry molecular orbitals, shown in Figure 2 with occupation numbers 1.94 and 0.32, respectively, do also contribute markedly to the planarity of the  cluster. These molecular orbitals sit on the virtual orbitals’ space in single reference methods, like the B3LYP, but enter into the valence orbitals when the static electron correlation is properly accounted for.

**Figure 3:**
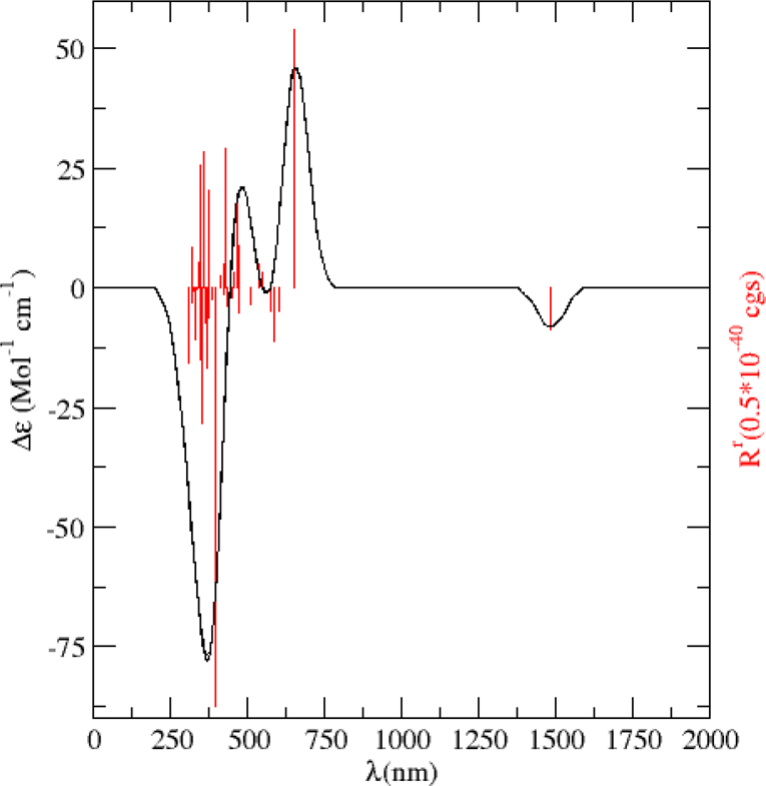
Simulated electronic circular dichroism (ECD) spectra of the of the *C*_2_ symmetry lowest energy triplet state ^3^B, of the  cluster. The 40 lowest electronic transitions were calculated, and the bandwidth used was σ = 0.2 eV.

For the triplet spin state of  we found a chiral isomer of *C*_2_ symmetry, with the ^3^B electronic state, to be the most stable isomer which turns out to be the only chiral isomer characterized for the cluster anion within an energy range of ∼30 kcal/mol. Boldyrev et al.^15^ characterized such a structure at the B3LYP/6-311+G* level of theory to lie 5.3 kcal/mol higher in energy than the most stable *C*_2*v*_(^1^A_1_), isomer. Our more refined calculations raise that energy difference up to 9.07 kcal/mol (see Table 2). This *C*_2_ symmetry lowest-lying triplet spin-state isomer must be optically active, and therefore, it could be proven with the aid of optical spectroscopy. In particular, if its electronic circular dichroism (ECD) spectrum could be recorded, differences in the UV–vis absorption spectrum between right- and left-circularly polarized light would reveal an excess of one enantiomer. This approach, which has became one of the most successful experimental means to unveil the chirality of a wide variety of (sub)nanostructures,^32^ has recently been suggested as possibly also being useful for the determination the chirality of small clusters.^33^ We are aware of the experimental difficulties associated with the recording of the gas-phase optical absorption spectra of mass- and charge-selected trapped cluster anions. Nevertheless, we provide herein the predicted ECD spectrum of the ^3^B electronic state of the lowest lying isomer of the triplet spin state of the  anionic cluster, see Figure 3, calculated at the B3LYP/aug-cc-pVTZ level of theory.

The most stable isomer of the boron pentamer cluster cation, , has a perfect pentagonal *D*_5*h*_ symmetrical structure, with a B–B bond distance of 1.550 Å. Indeed, this cluster shows no intra-annular boron–boron bonding. However, the lowest energy isomers of both, the triplet and quintet spin states have *C*_2*v*_ symmetry, featuring the three fused-triangles motif as do its parent neutral and anionic clusters irrespective of their spin states.

We have measured the electron delocalization of the lowest energy isomers of the  cluster’s singlet, triplet, and quintet spin states by means of three indices;^34,35^ i.e.: the multicenter normalized Giambiagi ring-current index, *I*_ring_, which quantifies the ring current along a given ordered set of atoms, the total number of delocalized electrons, denoted as , and the adjacent atom-pair electron delocalization index, δ(B,B′). All the above-mentioned indices have been calculated for the corresponding optimized MCSCF(4,15)/aug-cc-pVTZ corresponding multiconfigurational wave functions.

The δ(B,B′) value of 1.27 for the (D_5*h*_) ground state of  is indicative of a substantial electron delocalization among adjacent boron atoms. This is consistent with the total electron-pair delocalization which shows that the  delocalized electrons are delocalized among the five boron nuclei in two ways, nameley, 6.35 (5 × δ(B,B′)) electrons on the periphery, and the remaining 1.15 electrons, radially. The calculated value of the normalized Giambiagi index, I_ring_ = 15.6 × 10^–3^, reveals that the peripheral circular electron delocalization in the ground state of  is less than half of that of ground state of the classical  ring like anion,^35^ a fact that should be mainly ascribed to the larger diameter of the boron pentamer cation cluster.

Table 3 shows the values for the peripheral adjacent atoms-pair, δ(1), δ(2), δ(3), electron delocalization indices of the three symmetry independent bonds as shown in Figure 1.B, for the lowest energy isomers of the triplet and quintet spin states of . Having in mind that the total number of delocalized electrons for the triplet (see Table 3) is 6.25 electrons, we deduce that the intra-annular electron delocalization amounts 1.7 electrons. Comparing this number with that of the most stable (D_5*h*_) singlet spin state isomer (1.15 electrons, vide supra), puts forward the presence of substantial radial chemical bonding interactions in the triplet spin state isomer, as reflected by the adjacent intra-annular electron delocalization which amounts to 2 × δ(4) = 1.5, and leaves a tiny contribution of (1.7–1.5)/2 = 0.1 electrons for the nonadjacent intra-annular electron delocalization. Furthermore, the same reasoning yields 1.84 electrons for the adjacent intra-annular electron delocalization of the lowest energy isomer of the quintet spin state, which is concordance with its shorter interannular boron–boron distances, relative to the lowest energy isomer of the triplet spin state, as can be appreciated in Figure 1.

**Table 3:** Multicenter Normalized Giambiagi Ring-Current Index, *I*_ring_ (Multiplied by 10^3^), along with the Total, δ(B_5_), and Adjacent Atom-Pair Delocalization Indices, δ(B,B′), for the Symmetry Unique Bonds (See Figure 1B) of the Lowest Energy Structures of the  Clusters’ Singlet, Triplet, and Quintet Spin States and the Doublet and Quartet of the B_5_ Cluster^a^

state	*I*_ring_	δ(B_5_)	δ(1)	δ(2)	δ(3)	δ(4)
B_5_^+^						
^1^A_1_′(*D*_5*h*_)	15.6	7.50	1.27			
^3^B_2_(*C*_2*v*_)	1.6	6.25	0.80	0.85	1.25	0.75
^5^A_1_(*C*_2*v*_)	10.3	6.10	0.89	0.79	0.90	0.72
B_5_						
^2^B_2_(*C*_2*v*_)	4.8	7.15	1.09	1.05	1.22	0.67
^4^A′(*C*_*s*_)	4.5	7.15	(see Figure 4)			
B_5_^–^						
^1^A_1_(*C*_2*v*_)	–0.8	7.15	0.84	0.92	1.39	0.98
^3^B(*C*_2_)	1.5	7.43	1.07	1.10	1.26	0.76
^5^A_1_(*C*_2*v*_)	5.6	7.59	1.07	1.30	0.96	0.85

a All quantities are expressed in electrons.

**Figure 4:**
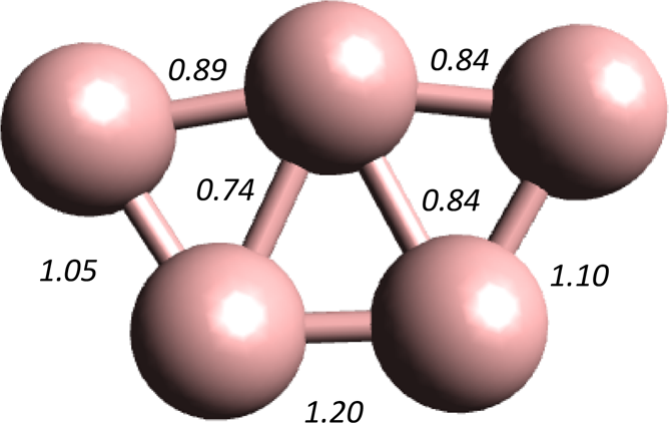
Adjacent atom-pair delocalization, δ(B,B′), indices for the lowest energy geometry of the B_5_ cluster’s *C*_*s*_ symmetry ^4^A′ state.

Analysis of delocalization indices of the neutral B_5_ lowest energy *C*_2*v*_ symmetry’s ^2^B_2_ electronic state shown in Table 3, reveals 5.5 electrons delocalized on the periphery and 1.65 electrons for the intra-annular electron delocalized. The lowest energy quartet spin state isomer is remarkable for the data of Figure 4 shows that it has 2.07 electrons delocalized intra-annularly and only 5.08 electrons on the periphery. This, along with the fact that adjacent intra-annular electron delocalization amounts to 1.58 electrons, leaves the non-negligible amount of 0.25 electrons for each of the two intra-annular nonadjacent electron delocalization indices.

Finally, the most stable singlet, triplet and quintet spin state isomers of , have 4.91, 5.60, and 5.70 electrons delocalized on their periphery and 2.24, 1,83 and 1.89 electrons delocalized intra-annularly, respectively (see Table 3).

Boldyrev et al. hypothesized^15^ that the complete delocalization of the valence π-type orbitals could contribute to the planarity of the boron clusters. This idea was formulated earlier in a different manner by Ugalde and Fowler,^9^ who argued that the planarity of the  cluster was largely due to the delocalization of π-type molecular orbitals, resembling benzene’s π molecular orbitals, which expand over the whole molecular plane. The present research reinforces Boldyrev’s hypothesis by bringing to the fore the existence of natural orbitals, radially delocalized on the central motif of the B_5_ clusters, which are reflected in the large values of the intra-annular electron delocalization indices found for all isomers characterized. This electron delocalization provides structural rigidity toward out-of-plane structural deformations.

### Dressed Boron Pentamer Clusters

The bonding of ligands to the atoms of planar bare clusters normally triggers a change of geometrical structure from planar to compact three-dimensional geometries, as recently documented by Walter et al.^18^ by means of large scale DFT calculations on a number of ligand-decorated clusters of precisely known composition and structure. The important lesson arising from their exhaustive analysis is that the jellium model^36,37^ can be used to account for the gained stability of the ligand-protected clusters. The key concept is that enhanced stability of the core cluster is achieved when the shell-closure condition, as dictated by the jellium model, is satisfied. In this respect, the role of the ligands can be circumscribed to modulating of the number of valence electrons of the core cluster confined by the jellium potential of the atomic cores of the cluster. The estimation of such a number is made using the following formula,
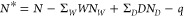
1where *N* is the number of valence electrons of the core cluster in the absence of the ligands, *N*_*W*_ is the number of electron withdrawing ligands each withdrawing *W* electrons, *N*_*D*_ is the number of electron donating ligands each donating *D* electrons, and *q* is the signed total charged of the ligand protected cluster. Then, the jellium model sets up an electrostatic energy functional for the interaction between valence electrons, whose electron density is ρ(**r**), and the cluster’s core ions, which is simply given by a Coloumbic term,
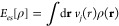
2where *v*_*j*_(*r*) is the spherically symmetric confinement potential generated by the cluster’s core ions positive charge distribution (”the jelly”) uniformly distributed in a sphere of radius *R*,
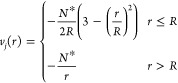
3with N* being the number of valence electrons confined by such potential. The spherical symmetry of the confining potential, *v*_*j*_(*r*), imposes the conservation of the angular momentum, and consequently, the resulting eigenfunctions can be classified in accordance to the angular momentum operators eigenfunctions, namely
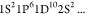
4where the superscripts stand for the maximum occupation of the corresponding orbital type. Developments of this model to account for confinement potentials coming from the core ions’ charge density distributions with symmetries other than spherical, specifically those reflecting polyhedral symmetries, which are particularly relevant for the present research, have been implemented in the DFT codes for any conceivable polyhedral shape of the background jellium density.^16^

We have searched for the optimum geometries of the singlet- and triplet-spin states of the boron pentamer hydride, [(BH)_5_]. The optimizations and subsequent harmonic vibrational frequency analysis of the stationary geometries found was performed by means of DFT calculations with the B3LYP approximate exchange-correlation hybrid functional and the aug-cc-PVTZ basis set. Energies were refined at the CCSD(T)/aug-cc-PVTZ level of theory.

**Figure 5:**
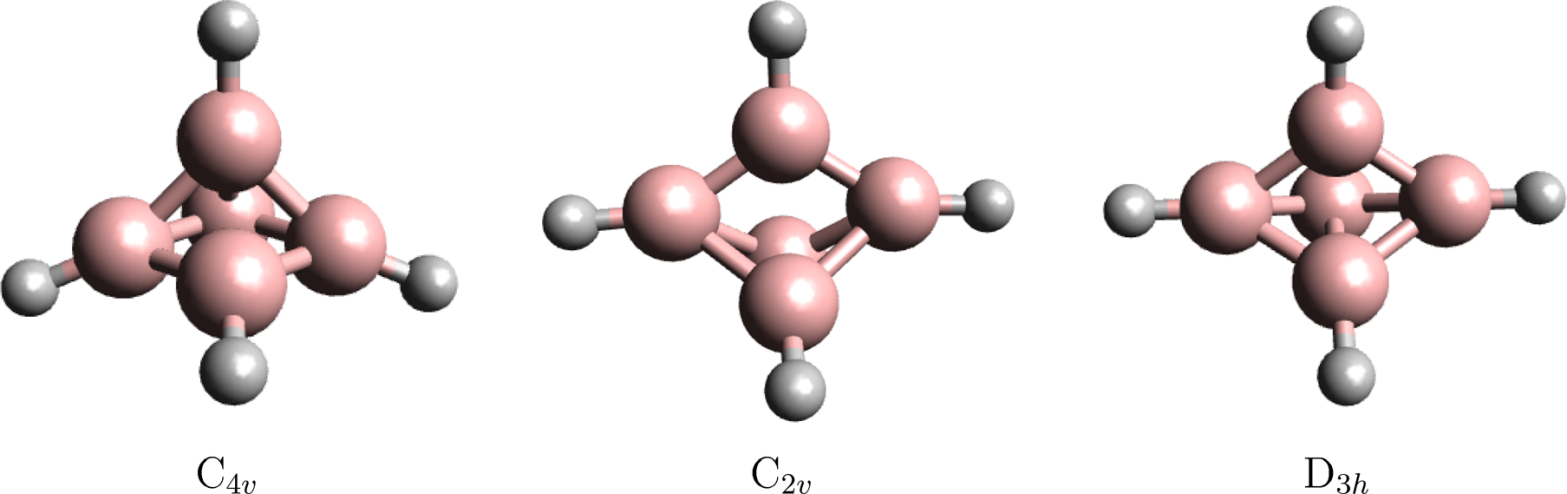
Geometries of the stationary B3LYP/aug-cc-pVTZ optimized structures of singlet and triplet pentamer boron hydridres, [(BH)_5_].

Three stationary points were characterized on both the singlet and triplet spin-state potential-energy surfaces. Figure 5 sketches the geometry of the optimized structures, the actual geometrical data along with their harmonic vibrational frequencies and their calculated T1-diagnostic values can found in the section 3 of the Supporting Information. Notice that the calculated T1-diagnostic values lie below the threshold value of 0.02, which is indicative of lack of (system-dependent) static electron correlation.^38^ Consequently, one must take care only of the remaining (universal) dynamic electron correlation. The latter is well accounted for by the single-reference gold-standard CCSD(T) method.^39^ Hence, the dressed clusters will be studied using the CCSD(T) single-reference wave function method on B3LYP optimized structures. The relative energies can be seen in Table 4.

**Table 4:** Relative B3LYP/aug-cc-pVTZ and CCSD(T)/aug-cc-pVTZ Energies with Zero Point Corrections Included of the Stationary Structures of the Singlet and Triplet Spin States of [(BH)_5_]^a^

singlet				triplet			
symm	NNFC	B3LYP	CCSD(T)	symm	NNFC	B3LYP	CCSD(T)
*C*_4*v*_	0	0.00	0.00	*D*_3*h*_	0	11.84	13.32
*C*_2*v*_	1	7.89	8.38	*C*_2*v*_	1	23.85	29.69
*D*_3*h*_	2	31.43	30.16	*C*_4*v*_	1	46.70	52.76

a NNFC stands for the number of negative force constants. All quantities are expressed in kcal/mol relative to the lowest, either B3LYP or CCSD(T), energy.

The lowest energy structure of the singlet-spin state potential energy surface has *C*_4*v*_ symmetry and ^1^A_1_ electronic state, and it corresponds to the most stable isomer of [(BH)_5_]. The next lowest energy lying structure has *C*_2*v*_ symmetry and features an isosceles trigonal bipyramid like geometry. However, it is a transition state rather than an stable isomer, as shown by its negative force constant of −0.1375 mdyn/Å. Finally, the highest energy singlet spin state isomer characterized has *D*_3*h*_ symmetry i.e.: an equilateral trigonal bipyramid geometry, but shows two negative force constants, −23.8300 and −0.1907 mdyn/Å.

The three stationary structures found on the triplet spin state potential energy surface have *D*_3*h*_, *C*_2*v*_, and *C*_4*v*_ symmetries, respectively. The *D*_3*h*_ symmetry one corresponds to the most stable triplet spin isomer and has ^3^A_1_′ electronic state. The *C*_2*v*_ symmetry stationary structure has the ^3^B_2_ electronic state, and corresponds to a transition state (it has one and only one negative force constant of −0.1022 mdyn/Å) between two equivalent *D*_3*h*_ minima. The *C*_4*v*_ symmetry stationary structure corresponds also to a transition state, as revealed by its calculated negative force constant of −0.2690 mdyn/Å.

Notice that most stable triplet state isomer is 13.32 kcal/mol less stable that the most stable singlet spin state isomer (see Table 4).

The geometry of the boron core cluster of the most stable isomer of the singlet spin state of the [(BH)_5_] cluster corresponds to a *nido*-borane type structure. This, in principle, contradicts Wade’s 4*n* rule.^40^ This is because the [(BH)_5_] cluster has 5 × 3 + 5 × 1 = 20 valence electrons which matches 4 × *n*, for *n* = 5. Thus, the predicted structure is a capped *closo*-borane, namely, a *n* – 1 vertex *closo*-polyhedron with one face capped. That is, a tetrahedron with one face capped. We found such a structure, but it turned out to have one negative force constant, indicating that it corresponds to a transition state, rather than to a stable structure. Furthermore, we also located a trigonal bipyramidal structure of *D*_3*h*_ symmetry, with two negative force constants, well above in energy with respect to the minimum energy *nido*-borane type isomer. Recall that a trigonal bipyramid, which corresponds to the *closo*-polyhedron of *n* = 5 vertices, is the predicted Wade optimum structure for boranes with 4 × *n* + 2 valence electrons, rather than for the 4 × *n* valence electrons that [(BH)_5_] has.

**Figure 6:**
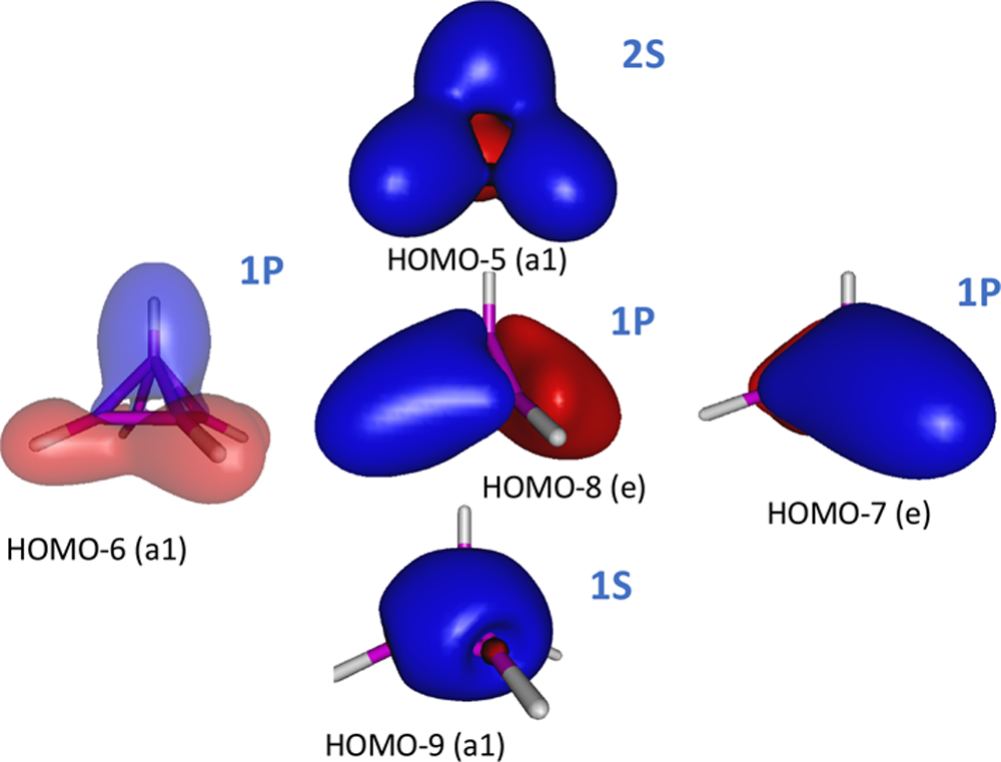
Valence molecular orbitals of the singlet spin *C*_4*v*_ symmetry’s ^1^A_1_ electronic state of [(BH)_5_] resembling the jellium model shell-closure molecular orbitals.

One way to understand our results, and put them in line with Wade’s 4*n* rule, is to start from the  cluster, which is well-known to possess a tetragonal pyramidal structure, as correctly predicted by Wade’s 4*n* rule. Namely, a *nido*-borane, as dictated by its 4 × *n* + 4 valence electrons. Now, let us look at this tetraanion cluster from the point of view of the jellium theory. Since the confinement potential has *C*_4*v*_ symmetry rather than spherical symmetry, the 1D orbitals must split accordingly, so that the shell closure attained by the 14 jellium valence electrons of  (see eq 1) is represented as , which accounts for its enhanced stability. Removal of the four 1D(e)^4^ electrons conserves the shell closure and accounts for the enhanced stability of the *C*_4*v*_ symmetry of the boron core cluster of the most stable singlet-spin state isomer of the [(BH)_5_] neutral cluster. Figure 6 shows the 1S^2^1P^6^2S^2^ occupied jellium molecular orbitals.

The remaining occupied HOMO–4 to HOMO molecular orbitals correspond the five B–H bonding orbitals.

**Figure 7:**
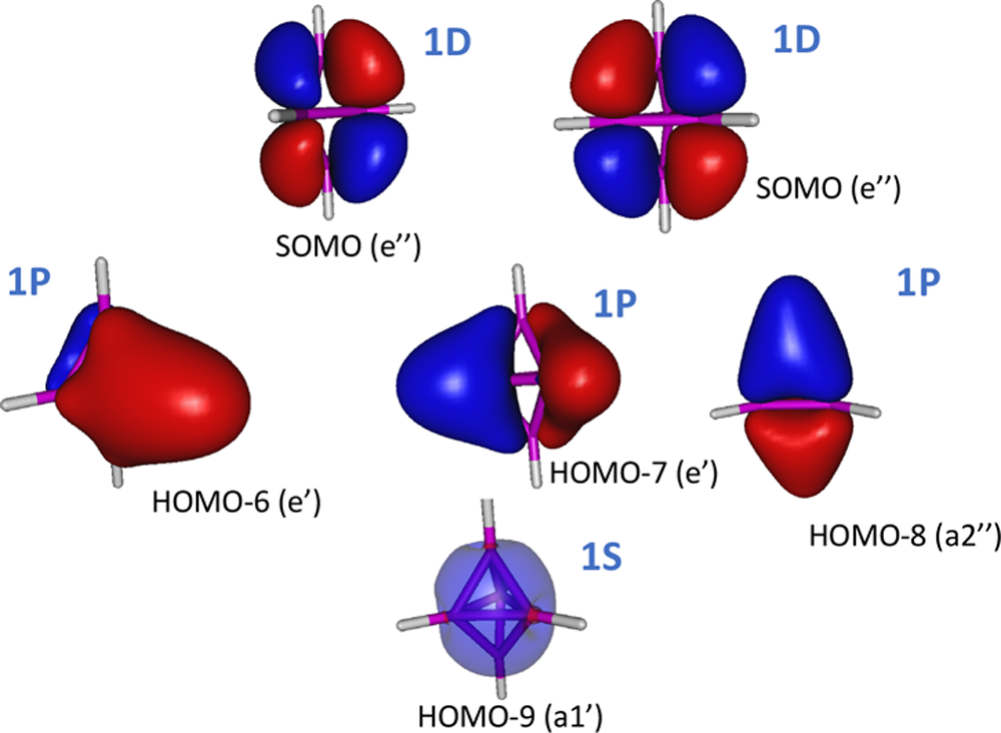
Valence molecular orbitals of the triplet spin *D*_3*h*_ symmetry’s ^3^A_1_′ electronic state of [(BH)_5_] resembling the jellium model shell-closure molecular orbitals.

The jellium electronic structure of the core boron cluster of triplet-spin state [(BH)_5_] can also be seen as arising from the well-known Wade rule satisfying trigonal bipyramidal  cluster.^41^ The confinement potential, which in this case has *D*_3*h*_ symmetry, splits the 1D orbitals into three sets, i.e., the doubly degenerated e″ and e′ symmetry sets and the a″ set. The trigonal bipyramidal  is singlet-spin-state stable because it achieves the  shell closure. Stripping off one electron from each of the two degenerate 1D(e″) orbitals leads to the orbital-occupation balanced stable triplet-spin state [(BH)_5_] cluster shown in Figure 7.

The remaining occupied HOMO–5 to HOMO–1 molecular orbitals correspond to the five B–H bonding orbitals.

**Figure 8:**
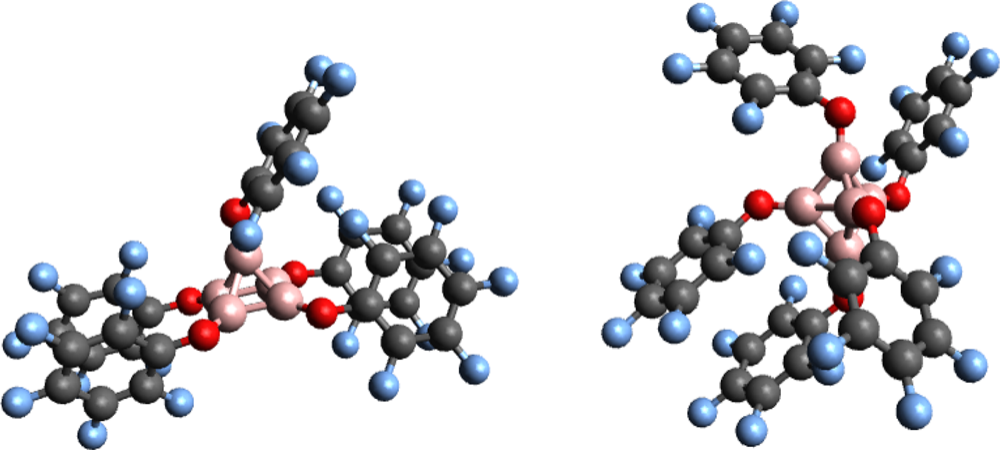
Optimum B3LYP-D3(BJ)/6-311++G** geometries of the singlet, on the left, and of the triplet, on the right, of the [(B(OR))_5_], R = −C_6_F_5_, clusters.

**Figure 9:**
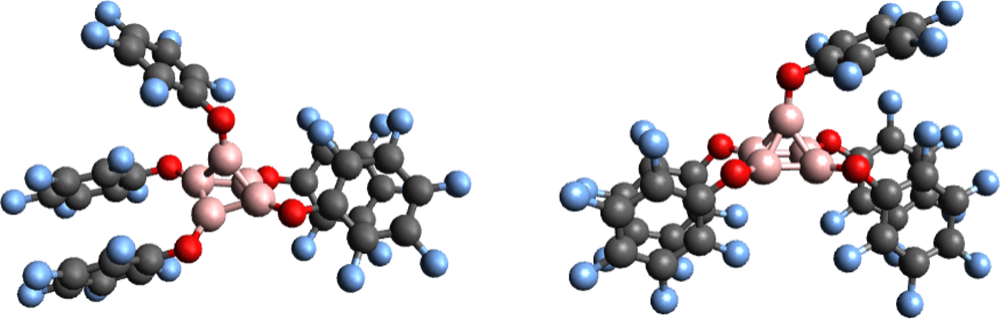
Optimum B3LYP-D3(BJ)/6-311++G** geometries of the doublet, on the left, and of the quartet, on the right, of the , R = −C_6_F_5_, clusters.

The robustness of structural motifs of the most stable isomers of both the singlet and triplet spin states remains for more voluminous ligands as shown in Figure 8, in spite of the deformation due to the π-stacking forces between the perfluorated benzene rings. The singlet–triplet energy difference raises up to 22.09 kcal/mol at the B3LYP-D3(BJ)/6-311++G** level of theory,^42^ which is to be compared with the corresponding relative energy of 11.84 kcal/mol for [(BH)_5_] (see Table 4).In this connection, we show in Figure 9, the B3LYP-D3(BJ)/6-311++G** optimum structures of the doublet and quartet spin states of the  pentaborate radical anions. The quartet spin state isomer is calculated to be 50.26 kcal/mol above the doublet one.

Given the structural stability, the large doublet-quartet energy gap, and the localization of the spin polarized radical electrons on the core boron pentamer cluster, which remains protected by the – OC_6_F_5_ ligands, these compounds may also be appropriate candidates to transfer spin polarization from the cluster’s electron radical to the ^19^F nuclei in dissolution dynamic nuclear polarization (D-DNP) experiments as demonstrated to occur for their parent dodecaborate radical anion,  clusters.^23^

## Conclusions

The geometry of the most stable isomers of bare boron small clusters of less that 15 atoms is known to be planar, a feature which has been rationalized in terms of their electronic structure. However, electronic structure calculations of small clusters may be plagued with a number of subtleties stemming from large static electron correlation. In such cases, it is unavoidable^43^ to carry out a high level of theory multiconfigurational type calculations, in order to address properly all features of their electronic structure and so establish a solid basis for further comparisons. Either the analysis of the weights of the various configurations of explicitly calculated MCSCF wave functions or the inspection of the T1-diagnostic values obtained from exploratory CCSD(T) calculations provides clear-cut clues as to whether such high level multiconfigurational type calculations are required.

Nevertheless, it must be made it clear that not always the higher level of theory calculations yield ”better” results that single reference calculations. Indeed, for the case of bare boron pentamer clusters the present exhaustive multiconfigurational type investigation reveals that single-reference methods are seen to be capable of finding all the low-lying stable structures of the  bare boron clusters, though their relative energies are found to differ by a few tens of kcal/mol, in the worst cases. Furthermore, it is a well-documented empirical fact of molecular electronic structure theory^44^ that as the number of electrons increases, static electron correlation effects decrease. Consequently, medium and large clusters are often amenable for single-reference descriptions. This is precisely what we have found and reported in the present study:  clusters require multireference wave functions, but B_5_(R)_5_ clusters do not.

The bare boron pentamer cluster stands prominently for it is known to be one of the so-called “magic number” clusters appearing in laser ablation experiments, a feature which pinpoints its remarkable stability. We have studied the most stable spin-state isomers of the cationic, neutral, and anionic boron pentamer clusters at the multiconfigutational self-consistent field supplemented with multiconfigutational quasi degenerate perturbation level of theory, which accurately accounts for both the static and dynamical electron correlation effects.

Our calculations show that the most stable isomer of the cationic species resembles a singlet spin-state perfect pentagon, with a bond length of 1.550 Å, which bears no intra-annular chemical bonding interactions, as shown by our analysis of the electron delocalization carried out in terms of the normalized Giambiagi ring-current index, and the total and adjacent atom-pair delocalization indices. The lowest energy triplet and quintet spin-state isomers have *C*_2*v*_ with large intra-annular chemical bonding interactions. This geometrical feature extends to both the neutral and the anionic species. Namely, the lowest-energy isomers of boron pentamer neutral and anionic cluster have peripheral and intra-annular sizable bonding interactions as revealed by the inspection of their corresponding valence natural orbitals. We found strong signatures of both π- and σ-type valence natural orbitals delocalized over the whole molecular plane, which impart great structural stability, as hypothesized earlier by Boldyrev et al.^15^ It is worth noting that, in accordance with our calculations, the lowest energy triplet spin-state isomer of the anionic boron pentamer cluster has *C*_2_ symmetry, and consequently, it should show optical activity.

Finally, we have studied the change of the structure of the cluster caused by the bonding of ligands to the boron atoms of the bare pentamer cluster, for its singlet and triplet spin-states. The former in found to be more stable than the latter by 13.32 kcal/mol. The geometrical structures of both spin-states lowest energy isomers can nicely be accounted for by considering the shell-closure of their delocalized valence orbitals as predicted by the jellium model extended to nonspherical confinement potentials and circumscribing the role of the ligand to modulate the total number of valence electrons assigned to the core cluster. This suggest that the jellium model could also be applicable and useful for the determination of both electronic and geometrical features of nonmetallic clusters.

## Supporting Information Available

Coordinates, energies, ZPVE, and frequencies of the studied structures (PDF)
