# Supplementary material for: Electronic Structure and Electron Delocalization in Bare and Dressed Boron Pentamer Clusters
Source: J Phys Chem A. 2021 Jun 11;125(24):5246–55. doi: 10.1021/acs.jpca.1c02305 (PMC9159651; doi:10.1021/acs.jpca.1c02305)
Supplement: Supplementary file 1 — jp1c02305_si_001.pdf [file jp1c02305_si_001.pdf]

Supporting Information: Electronic Structure and Electron Delocalization in Bare and Dressed Boron Pentamer Clusters

Jose M. Mercero * and Jesus M. Ugalde

Kimika Fakultatea, Euskal Herriko Unbertsitatea (UPV/EHU), and Donostia International Physics Center (DIPC), P.K. 1072, 20080 Donostia, Euskadi, Spain

E-mail: jm.mercero@ehu.eus

Contents

1 Selection of the Complete Active Space Molecular Orbitals for the Multi-configurational Self-Consistent Field Calculations S-3

2 Coordinates, energies, ZPVE and Frequencies of $B_5^{+1,0,-1}$ S-5

2.1 B_5^+ ($^1A_1'$, D_{5h}) S-5

2.2 B_5^+ ($^3B_2$, C_{2v}) S-6

2.3 B_5^+ ($^5A_1$, C_{2v}) S-6

2.4 B_5 ($^2B_2$, C_{2v}) S-7

2.5 B_5 ($^4A_1'$, C_s) S-8

2.6 B_5^- ($^1A_1'$,C_{2v}) S-8

2.7 B_5^- (^3B, C_2) S-9

2.8 B_5^- ($^5A_1'$,C_2) S-10

3 Coordinates, energies, ZPVE and Frequencies of B_5H_5 S-10

3.1 B_5H_5 ($^1A_1$, C_{4v}) S-10

3.2 B_5H_5 ($^1A_1$, C_{2v}) S-12

3.3 B_5H_5 ($^1A_1'$, D_{3h}) S-13

3.4 B_5H_5 ($^3A_1'$, D_{3h}) S-14

3.5 B_5H_5 ($^3B_2$, C_{2v}) S-16

3.6 B_5H_5 ($^3A_1$, C_{4v}) S-17

4 Coordinates, energies, ZPVE and Frequencies of B_5R_5, R=–C_6F_5 S-18

4.1 B_5R_5, R=-C_6F_5, (^1A, C1) S-18

4.2 B_5R_5, R=-C_6F_5, (^3A, C1) S-26

4.3 $B_5R_5{}^-$, R=-C_6F_5 (^2A, C1) S-33

4.4 $B_5R_5{}^-$, R=-C_6F_5 (^4A, C1) S-40

1 Selection of the Complete Active Space Molecular Orbitals for the Multiconfigurational Self-Consistent Field Calculations

The reasoning for the selection of our Complete Active Space (CAS) for the calculation of the reference MCSCF wave function is based on the Dixon's analysis of the valence molecular in annular ring-like molecules made of main-group electron-deficient atoms [1]. Dixon has established that p-type atomic orbitals do conform the active space which carries static electron correlation, and the s-type atomic orbitals mainly contribute dynamic electron correlation. Thus, we have 5×3 p-type atomic orbital which generate our 15 molecular orbitals of the CAS. For the neutral boron pentamer cluster we have 5 electrons in these orbitals, since each boron atoms contributes one p-type electrons. Cationic and anionic pentamer clusters follow suit. Subsequently, multi-reference quasi-degenerate perturbation, MCDQPT, calculations have been carried out correlating all core and valence s-type atomic orbitals of the boron atoms. This brings in the missed dynamical electron correlation in the reference MCSCF wave function. Notice that we provided energies at the MCQDPT level of theory, not at the MCSCF, which is used exclusively to set a reference wave function for the MCDQPT calculations.

We have compared, nonetheless, our MCSCF calculations with other similar ones found in the open chemical literature. In particular Zhai et al. [2] reported MCSCF(8,8)/6-311+G* and MCSCF(7,8)/6-311+G* calculations on the B_5^- and B_5 clusters, respectively. Their results and ours for the optimized geometries and harmonic vibrational frequencies are shown in Table S1 below.

[1]Truong Ba Tai, Daniel J. Grant, Minh Tho Nguyen, and David A. Dixon. Thermochemistry and Electronic Structure of Small Boron Clusters ($B_n, n = 5 - 13$) and Their Anions. *J. Phys. Chem. A.*, 114:994–1007, 2010

[2]Hua-Jin Zhai, Lai-Sheng Wang, Anastassia N. Alexandrova, and Alexander I. Boldyrev. Electronic Structure and Chemical Bonding of B_5 and B_5^- by Photoelectron Spectroscopy and ab initio Calculations. *J. Chem. Phys.*, 117:7917–7923, 2002.

Table S1: MCSCF(N_1,8)/6-311+G* and MCSCF(N_2,15)/aug-cc-pTZVP optimum bond lengths, in Å, and hamonic vibrational frequencies, in cm^{-1}, for the B_5^- (N_1=8, N_2=6) and B_5(N_1=7, N_2=5) clusters.

	B_5^-		B_5	
	MCSCF(8,8)/ 6-311+G*	MCSCF(6,15)/ aug-cc-PVTZ	MCSCF(7,8)/ 6-311+G*	MCSCF(5,15)/ aug-cc-PVTZ
R(B1–B2,3)	1.752	1.749	1.868	1.871
R(B1–B4,5)	1.632	1.615	1.584	1.567
R(B2–B3)	1.557	1.555	1.552	1.546
R(B2–B4)	1.583	1.575	1.591	1.589
$\omega_1(a_1)$	1320	1316	1337	1379
$\omega_2(a_1)$	985	983	958	974
$\omega_3(a_1)$	738	736	759	782
$\omega_4(a_1)$	647	673	596	600
$\omega_5(a_2)$	402	385	368	361
$\omega_6(b_1)$	227	272	306	269
$\omega_7(b_2)$	1121	1316	1170	1166
$\omega_8(b_2)$	994	1153	939	970
$\omega_9(b_2)$	637	630	527	453

The agreement between the data of the calculations carried our with the two different CAS's and basis functions is remarkable and suggestive of the equivalence, in chemical terms, of the two approaches.

2 Coordinates, energies, ZPVE and Frequencies of $B_5^{+1,0,-1}$

Data obtained at the MCSCF(N,15)/aug-cc-pVTZ level of theory, where N=4 for the cation, N=5 for the neutral and N=6 for the anion.

2.1 B_5^+ ($^1A_1'$, D_{5h})

```
B        0.0000000000  -1.2539203661   0.4074227528
B        0.0000000000   1.2539203670   0.4074227560
B        0.0000000000  -0.7749649936  -1.0666476505
B        0.0000000000   0.7749649831  -1.0666476244
B        0.0000000000   0.0000000096   1.3184497662
```

```
     FREQUENCIES IN CM**-1, IR INTENSITIES IN DEBYE**2/AMU-ANGSTROM**2,
       FREQUENCY:        12.58        7.48        6.48        0.27       25.87
    IR INTENSITY:      0.00000     0.00000     0.00000     0.00000     0.00000
       FREQUENCY:        26.12      144.71      150.97      317.34      317.40
    IR INTENSITY:      0.00000     0.00001     0.00000     0.00000     0.00000
       FREQUENCY:      1004.42     1117.90     1119.35     1425.39     1426.27
    IR INTENSITY:      0.00001     2.85079     2.87888     0.00004     0.00012
```

E(MCSCF) -122.9590887138 A.U.

ZPVE 0.016001 A.U.

E(MCQDPT)= -123.3705890242 A.U.

2.2 B_5^+ ($^3B_2$, C_{2v})

B	0.4798466442	1.1235421975	0.0002821043
B	-1.0388517808	0.6442128240	0.0001967870
B	-1.3364470560	-0.9040516353	-0.0009149873
B	0.2814470232	-0.8911428767	0.0013904849
B	1.6140051695	0.0274394905	-0.0009543890

FREQUENCIES IN CM**-1, IR INTENSITIES IN DEBYE**2/AMU-ANGSTROM**2,

FREQUENCY:	27.80	14.48	9.81	12.74	26.01
IR INTENSITY:	0.04592	0.00881	0.00178	0.01893	0.00038
FREQUENCY:	57.39	249.01	333.26	341.19	460.92
IR INTENSITY:	0.05382	0.52241	0.00290	4.08679	0.27472
FREQUENCY:	785.16	862.87	927.47	1072.97	1321.06
IR INTENSITY:	0.85197	1.63428	0.35770	0.95491	0.00131

E(MCSCF) -122.8961519740 A.U.

ZPVE 0.014475 A.U.

E(MCQDPT)= -123.3348210281 A.U.

2.3 B_5^+ ($^5A_1$, C_{2v})

B	5.0	-0.0011054940	0.7467372300	-0.7274193099
B	5.0	-0.0015881798	-0.7469869741	-0.7265050828
B	5.0	0.0026960927	-1.9266623451	0.3466860676
B	5.0	0.0017256142	1.9248511027	0.3499592956

B	5.0	-0.0017280331	0.0020609865	0.7572790294

FREQUENCIES IN CM**-1, IR INTENSITIES IN DEBYE**2/AMU-ANGSTROM**2,

FREQUENCY:	18.47	7.11	5.38	8.33	16.87
IR INTENSITY:	0.00416	0.00919	0.00097	0.00349	0.06645
FREQUENCY:	27.64	166.79	216.06	244.15	344.53
IR INTENSITY:	0.01200	0.00446	0.00938	0.00001	0.75900
FREQUENCY:	726.63	735.50	1005.63	1018.06	1527.67
IR INTENSITY:	0.30228	0.00908	0.23316	17.68851	1.58758

E(MCSCF) -122.8217284981 A.U.

ZPVE 0.013635 A.U.

E(MCQDPT)= -123.3089918367 A.U.

2.4 B_5 ($^2B_2$, C_{2v})

B	5.0	0.0000000000	-0.7745280582	0.2663988761
B	5.0	0.0000000000	1.0881466981	0.4432597171
B	5.0	-0.0000000000	-0.9541436236	-1.3129364026
B	5.0	-0.0000000000	0.5849544256	-1.0184682433
B	5.0	-0.0000000000	0.0555705582	1.6217460823

FREQUENCY:	0.00	0.00	0.00	0.00	0.00
IR INTENSITY:	0.00333	0.00320	0.00255	0.00640	0.00371
FREQUENCY:	0.00	269.87	361.14	456.42	599.86
IR INTENSITY:	0.00594	0.27466	0.00000	0.81374	0.22063
FREQUENCY:	782.60	973.10	974.76	1166.65	1379.72
IR INTENSITY:	0.05632	0.15654	0.05642	0.17457	0.16659

E(MCSCF) -123.2390964208 A.U.

ZPVE 0.015865 A.U.

E(MCQDPT)= -123.7006631774 A.U.

2.5 B_5 ($^4A_1'$, C_s)

B	-0.4472792633	-1.0617506145	0.2008014655
B	-0.0696827792	1.4715930691	-0.9873779597
B	0.7180343284	-0.9147286791	1.3075949675
B	0.5310493839	0.4116004366	0.2894895704
B	-0.7321216700	0.0932857879	-0.8105080437

FREQUENCIES IN CM**-1, IR INTENSITIES IN DEBYE**2/AMU-ANGSTROM**2,

FREQUENCY:	0.00	0.00	0.00	0.00	0.00
IR INTENSITY:	0.00022	0.00042	0.00039	0.00218	0.00213
FREQUENCY:	0.00	220.43	293.72	452.84	503.94
IR INTENSITY:	0.00020	0.08245	0.08784	0.57355	0.63082
FREQUENCY:	616.55	913.94	950.98	1131.19	1429.24
IR INTENSITY:	0.17219	0.62089	0.44432	5.91605	0.77388

E(MCSCF) -123.1693538327 A.U.

ZPVE 0.01483 A.U.

E(MCQDPT)= -123.6504109063 A.U.

2.6 B_5^- ($^1A_1'$,C_{2v})

B	0.0000000000	-0.6752835841	0.2318199527
B	0.0000000000	1.0593918218	0.4583512844
B	-0.0000000000	-0.9918148462	-1.3603636541
B	-0.0000000000	0.5525173123	-1.0123685717

B -0.0000000000 0.0551892962 1.6825610182

FREQUENCIES IN CM**-1, IR INTENSITIES IN DEBYE**2/AMU-ANGSTROM**2,

FREQUENCY:	0.00	0.00	0.00	0.00	0.00
IR INTENSITY:	0.00348	0.03190	0.01446	0.00035	0.02576
FREQUENCY:	0.00	272.25	385.06	623.30	666.47
IR INTENSITY:	0.00875	1.39943	0.00018	1.71918	0.13783
FREQUENCY:	736.76	983.76	1030.29	1153.78	1316.45
IR INTENSITY:	0.49614	0.01730	0.79446	0.04319	1.30469

E(MCSCF) -123.3022749379 A.U.

ZPVE 0.016330 A.U.

E(MCQDPT)= -123.7968709 A.U.

2.7 B_5^- (^{3}B, C_2)

B	0.4678991443	1.0488944611	0.1468210203
B	-0.9847907750	0.5878902133	-0.1592856878
B	-1.3682462550	-0.9209675372	0.0826628494
B	0.2358389461	-0.7466712459	0.0058323089
B	1.6492989398	0.0308541087	-0.0760304909

FREQUENCIES IN CM**-1, IR INTENSITIES IN DEBYE**2/AMU-ANGSTROM**2,

FREQUENCY:	7.88	2.40	0.49	7.18	17.43
IR INTENSITY:	0.00032	0.00033	0.00000	0.00215	0.00018
FREQUENCY:	50.71	305.31	372.91	553.21	620.59
IR INTENSITY:	0.00489	0.09695	0.00448	0.05687	0.67215
FREQUENCY:	707.17	956.99	1018.96	1175.33	1177.01
IR INTENSITY:	0.07332	0.17363	0.62715	1.38094	1.12071

E(MCSCF) -123.2832807125 A.U.

ZPVE 0.015691 A.U.

E(MCQDPT)= -123.7817829256 A.U.

2.8 B_5^- ($^5A_1^{'}$,C_2)

B	-0.0001067338	-0.8047661803	-0.7770817113
B	0.0002022938	0.8128787804	-0.8385471738
B	0.0001363163	1.7348919873	0.4264240457
B	-0.0000579629	-1.7366706434	0.4041159589
B	-0.0001739133	-0.0063339440	0.7004659781

FREQUENCIES IN CM**-1, IR INTENSITIES IN DEBYE**2/AMU-ANGSTROM**2,

FREQUENCY:	0.00	0.00	0.00	0.00	0.00
IR INTENSITY:	0.00023	0.00022	0.00006	0.00014	0.00018
FREQUENCY:	0.00	306.04	330.84	436.41	448.24
IR INTENSITY:	0.00018	0.02061	0.19997	0.00694	0.07089
FREQUENCY:	629.94	827.07	933.20	1171.80	1379.82
IR INTENSITY:	0.77002	0.12026	0.16714	0.63086	1.00239

E(MCSCF) -123.2220705866 A.U.

ZPVE 0.014725 A.U.

E(MCQDPT)= -123.7489102501 A.U.

3 Coordinates, energies, ZPVE and Frequencies of B_5H_5

Data calculated at the B3LYP/aug-cc-pVTZ level of theory.

3.1 B_5H_5 ($^1A_1$, C_{4v})

B	0.000000	0.000000	0.999291

B	0.000000	1.194185	-0.220226
B	1.194185	-0.000000	-0.220226
B	-1.194185	0.000000	-0.220226
B	-0.000000	-1.194185	-0.220226
H	0.000000	2.272640	-0.695033
H	2.272640	-0.000000	-0.695033
H	-2.272640	0.000000	-0.695033
H	-0.000000	-2.272640	-0.695033
H	0.000000	0.000000	2.188190

Harmonic frequencies (cm**-1), IR intensities (KM/Mole)

Frequencies --	274.9549	444.7731	444.7731
IR Inten --	0.0000	38.0304	38.0304
Frequencies --	550.1294	565.2993	585.2936
IR Inten --	0.9734	0.0000	0.0000
Frequencies --	655.9775	655.9775	705.2727
IR Inten --	72.6442	72.6442	16.2287
Frequencies --	705.2727	785.2764	804.3971
IR Inten --	16.2287	0.0000	19.4350
Frequencies --	814.9108	817.4176	834.0212
IR Inten --	0.0000	0.0000	10.8522
Frequencies --	834.0212	973.4749	1035.3157
IR Inten --	10.8522	3.7089	4.7819
Frequencies --	1035.3157	2620.8883	2713.6768
IR Inten --	4.7819	177.2971	0.0000
Frequencies --	2721.9765	2721.9765	2733.9956
IR Inten --	67.8776	67.8776	3.8515

E(B3LYP)	-127.2006159 A.U.
ZPVE	0.061589 A.U.
E(CCSD(T))	-126.8317001 A.U.
T1 Diagnostic	0.01506980

3.2 B_5H_5 ($^1A_1$, C_{2v})

B	0.000000	-0.000000	0.902078
B	1.326905	0.000000	0.000000
B	-1.326905	-0.000000	0.000000
B	-0.000000	-0.796322	-0.907353
B	-0.000000	0.796322	-0.907353
H	2.499849	0.000000	-0.065896
H	-2.499849	-0.000000	-0.065896
H	-0.000000	-1.817898	-1.502915
H	-0.000000	1.817898	-1.502915
H	0.000000	-0.000000	2.091748

```
Harmonic frequencies (cm**-1), IR intensities (KM/Mole)
 Frequencies --   -270.9179              378.1441              425.4589
 IR Inten    --      0.3902                5.0999                0.0000
 Frequencies --    515.2577              524.1403              566.9600
 IR Inten    --     19.6283                0.0275               17.8165
 Frequencies --    593.4341              607.1272              672.7890
 IR Inten    --      9.6240                0.3355                7.2808
 Frequencies --    747.4116              783.3109              794.9449
 IR Inten    --     91.9566                0.0000                9.4792
```

Frequencies --	812.3467	839.9223	847.3631
IR Inten --	8.8883	2.4546	4.8675
Frequencies --	886.0431	973.9954	1026.6442
IR Inten --	0.0000	6.4409	123.9850
Frequencies --	1103.0330	2621.4388	2672.2025
IR Inten --	0.0010	20.3301	126.6021
Frequencies --	2694.3536	2758.0266	2762.2880
IR Inten --	60.0845	161.3131	0.0727

E(B3LYP)	-127.187068 A.U.
ZPVE	0.060614 A.U.
E(CCSD(T))	-126.8173579 A.U.
T1 Diagnostic	0.01424297

3.3 B_5H_5 ($^1A_1'$, D_{3h})

B	0.000000	0.000000	1.043315
B	1.350527	0.000000	0.000000
B	-1.350527	0.000000	0.000000
B	0.000000	0.903537	-0.521657
B	0.000000	-0.903537	-0.521657
H	2.522360	0.000000	0.000000
H	-2.522360	0.000000	0.000000
H	0.000000	0.000000	2.226277
H	0.000000	1.928013	-1.113139
H	0.000000	-1.928013	-1.113139

Harmonic frequencies (cm**-1), IR intensities (KM/Mole)

Frequencies --	-2051.6970	-478.7044	339.4093

IR Inten	--	4555.9740	194.3897	0.1815
Frequencies	--	367.8791	480.7071	613.1506
IR Inten	--	0.0000	0.3594	1.4002
Frequencies	--	615.3920	615.5250	639.6977
IR Inten	--	17.1724	0.0000	0.0029
Frequencies	--	687.7517	721.5126	756.8970
IR Inten	--	0.5994	2.7207	1.9839
Frequencies	--	762.1267	784.0574	834.6342
IR Inten	--	0.0000	15.0103	78.4585
Frequencies	--	869.2350	895.4422	934.1546
IR Inten	--	0.7014	1.0800	0.0352
Frequencies	--	964.3666	2668.4913	2671.6396
IR Inten	--	0.1987	77.4579	124.3653
Frequencies	--	2687.9392	2777.7087	2785.1075
IR Inten	--	0.6380	113.6184	0.0000

E(B3LYP)	-127.1469618 A.U.
ZPVE	0.058031 A.U.
E(CCSD(T))	-126.780083 A.U.
T1 Diagnostic	0.01636528

3.4 B_5H_5 ($^3A_1'$, D_{3h})

B	-0.000000	0.000000	1.044449
B	1.342805	0.000000	0.000000
B	-1.342805	0.000000	0.000000
B	-0.000000	0.904520	-0.522225
B	-0.000000	-0.904520	-0.522225
H	2.515602	0.000000	0.000000

H	-2.515602	0.000000	0.000000
H	-0.000000	0.000000	2.227592
H	-0.000000	1.929151	-1.113796
H	-0.000000	-1.929151	-1.113796

Harmonic frequencies (cm**-1), IR intensities (KM/Mole)

Frequencies --	410.5336	451.5838	451.5840
IR Inten --	93.8325	0.7492	0.7492
Frequencies --	523.2006	523.2036	602.9046
IR Inten --	0.0000	0.0000	1.6031
Frequencies --	602.9051	687.3433	687.3438
IR Inten --	1.6033	0.0000	0.0000
Frequencies --	697.8579	697.8580	700.6069
IR Inten --	3.4401	3.4406	0.0000
Frequencies --	732.1323	732.1325	764.2678
IR Inten --	0.0000	0.0000	0.0000
Frequencies --	794.1897	880.5531	880.5531
IR Inten --	92.6045	0.0080	0.0080
Frequencies --	948.1574	2667.9118	2667.9118
IR Inten --	0.0000	114.1864	114.1866
Frequencies --	2685.7496	2769.7692	2776.6259
IR Inten --	0.0000	122.4419	0.0000

E(B3LYP)	-127.1801558 A.U.
ZPVE	0.060000 A.U.
E(CCSD(T))	-126.8088704 A.U.
T1 Diagnostic	0.02245587

3.5 B_5H_5 ($^3B_2$, C_{2v})

B	0.000000	0.000000	0.913236
B	0.000000	1.346731	-0.052580
B	1.075902	-0.000000	-0.363136
B	-1.075902	0.000000	-0.363136
B	-0.000000	-1.346731	-0.052580
H	0.000000	2.518480	-0.109997
H	1.951640	-0.000000	-1.149663
H	-1.951640	0.000000	-1.149663
H	-0.000000	-2.518480	-0.109997
H	0.000000	0.000000	2.110303

Harmonic frequencies (cm**-1), IR intensities (KM/Mole), Raman scattering

Frequencies --	-406.4814	191.7140	295.0795
IR Inten --	0.0000	15.2725	0.0000
Frequencies --	297.5712	377.1868	484.4164
IR Inten --	12.6966	0.0515	31.5955
Frequencies --	522.6480	563.1895	627.3723
IR Inten --	1.9274	0.0013	11.3069
Frequencies --	649.7239	679.2648	699.4414
IR Inten --	1.0672	3.1610	14.0015
Frequencies --	729.6678	759.4423	775.2459
IR Inten --	0.3999	0.0000	2.6309
Frequencies --	800.1267	908.2363	970.4648
IR Inten --	0.7079	0.7890	0.0594
Frequencies --	1059.9593	2529.8344	2728.9857
IR Inten --	8.1703	0.3084	12.6969
Frequencies --	2735.2912	2768.8982	2769.6481

IR Inten --	5.3051	76.4219	1.7883

E(B3LYP)	-127.1577986 A.U.
ZPVE	0.0567800 A.U.
E(CCSD(T))	-126.7795688 A.U.
T1 Diagnostic	0.01926941

3.6 B_5H_5 ($^3A_1$, C_{4v})

B	0.000000	0.000000	1.062993
B	1.268623	-0.000000	0.000000
B	-0.000000	-1.268623	0.000000
B	0.000000	1.268623	0.000000
B	-1.268623	0.000000	0.000000
H	2.387544	-0.000000	-0.360259
H	-0.000000	-2.387544	-0.360259
H	0.000000	2.387544	-0.360259
H	-2.387544	0.000000	-0.360259
H	0.000000	0.000000	2.265968

Harmonic frequencies (cm**-1), IR intensities (KM/Mole)

Frequencies --	-338.0561	325.9676	382.2426
IR Inten --	12.1093	0.0245	5.1786
Frequencies --	397.0267	488.2074	505.6288
IR Inten --	59.9031	0.2408	0.0000
Frequencies --	574.3620	631.5765	634.7496
IR Inten --	10.6075	42.7426	10.6716
Frequencies --	645.4713	722.7432	739.6771
IR Inten --	5.4255	4.9233	1.6302
Frequencies --	741.2885	782.3256	788.5148

IR Inten	--	5.0064	0.7010	0.0000
Frequencies	--	953.0104	976.3020	1020.2177
IR Inten	--	0.5626	1.3423	8.9022
Frequencies	--	2400.1875	2474.1917	2738.7680
IR Inten	--	0.0000	0.9193	2.8573
Frequencies	--	2739.3649	2754.5917	2758.2221
IR Inten	--	62.3685	37.1928	8.8055

E(B3LYP)	-127.1265194 A.U.
ZPVE	0.061908 A.U.
E(CCSD(T))	-126.7479256 A.U.
T1 Diagnostic	0.02046609

4 Coordinates, energies, ZPVE and Frequencies of B_5R_5, R=–C_6F_5

Data obtained at the B3LYP-D3(BJ)/6-311++G** level of theory.

4.1 B_5R_5, R=-C_6F_5, (^{1}A, C1)

C	3.4480005762	-1.1974818117	2.5118489149
C	2.7926031823	-0.2912001217	1.6782283967
C	3.5677880596	0.5238975484	0.8553282073
C	4.9526977215	0.4485446845	0.8594392306
C	5.590970345	-0.456120944	1.6993341201
C	4.8355004949	-1.2809413619	2.5254089344
O	1.4431679267	-0.1742638578	1.7443325089
B	0.5750782342	-0.3265997984	0.6857208509

B	0.5567818685	-0.9355144412	-0.8901213137
O	1.4008106577	-1.4558705072	-1.8352875173
C	2.7470671832	-1.5645725878	-1.672411496
C	3.6029467875	-0.7804260126	-2.4435828166
C	4.981846381	-0.9266831246	-2.349830768
C	5.5206232269	-1.8624033631	-1.4740286288
C	4.6784073931	-2.6437846166	-0.6914337944
C	3.3024036051	-2.4903231363	-0.7916715527
F	3.0919808771	0.1291431412	-3.2759020314
F	5.7877646218	-0.1649541018	-3.0882893368
F	6.8423214639	-1.9966190587	-1.3683939586
F	5.1943479127	-3.5237736195	0.1697927661
F	2.4967029942	-3.2343991809	-0.0237194124
F	2.9629805123	1.4082459431	0.0405022959
F	5.6679757604	1.2325723499	0.04910697
F	6.9203812827	-0.5427831579	1.6997339478
F	5.4436745339	-2.1563135891	3.3234596578
F	2.7358038134	-1.9963349975	3.3058518447
B	-0.2748118998	0.6084857986	-0.562818999
O	-0.2689165676	1.8253902701	-1.1172381968
C	0.4704915076	2.9133767825	-0.739397223
C	1.2780430382	3.5384662032	-1.6847588751
C	2.0392838152	4.6463468914	-1.3342487714
C	1.993545681	5.1364742927	-0.0336243888
C	1.1850469613	4.5198142949	0.9145077063
C	0.4244210091	3.414371038	0.5578424105
F	1.3379799343	3.0580408934	-2.9255795961

F	2.8186956994	5.2350003332	-2.2400102467
F	2.7256896784	6.1963824201	0.3046882981
F	1.1408646964	4.9882180741	2.1615668392
F	-0.3500455345	2.8167555907	1.4683325347
B	-1.1272842025	-0.272610477	0.7312020444
O	-1.959485134	0.0102210365	1.7802038148
C	-3.3028484974	-0.1994578941	1.771298593
C	-4.1713037763	0.8835460622	1.8938972589
C	-5.5461661365	0.6901203802	1.9567795582
C	-6.0689173113	-0.5966449811	1.8950839486
C	-5.214132838	-1.6853034874	1.7679647703
C	-3.8425090342	-1.4825235919	1.7060629759
F	-3.6782895367	2.1225099674	1.9431581196
F	-6.3646948758	1.7358652835	2.0659083345
F	-7.3873131694	-0.7863915671	1.9405266905
F	-5.7139461323	-2.9204913584	1.6860196137
F	-3.0247747118	-2.5349897978	1.5718687791
B	-1.1465365901	-0.9127251794	-0.8337581494
O	-2.0291018988	-1.5127672358	-1.7031858901
C	-3.3796917425	-1.4861011015	-1.5779005302
C	-4.0956677766	-2.6824719673	-1.6153725424
C	-5.4843335967	-2.6858027416	-1.5580196999
C	-6.1789596673	-1.4856965882	-1.4584197483
C	-5.4796201222	-0.2854931085	-1.417298557
C	-4.0935269511	-0.2919072264	-1.4794942659
F	-3.4411124952	-3.840618337	-1.7013300414
F	-6.1515345967	-3.8384971093	-1.5874021816

```
 F   -7.5099419295   -1.4851302985   -1.3870549372
 F   -6.1396794744   0.8698997108   -1.3041184594
 F   -3.4301232376   0.8750024476   -1.4446100104
```

```
Harmonic frequencies (cm**-1), IR intensities (KM/Mole)
 Frequencies --      7.3357                 10.3347                 12.6759
 IR Inten    --      0.0008                  0.0099                  0.0083
 Frequencies --     14.7688                 16.8558                 21.9023
 IR Inten    --      0.0341                  0.0859                  0.0668
 Frequencies --     26.3174                 30.7699                 34.0456
 IR Inten    --      0.0744                  0.1364                  0.5093
 Frequencies --     38.5366                 39.2378                 41.2139
 IR Inten    --      0.0462                  0.2850                  0.1208
 Frequencies --     42.4461                 56.5538                 63.4347
 IR Inten    --      0.0216                  0.0008                  0.1198
 Frequencies --     65.0300                 72.2431                 74.8794
 IR Inten    --      0.0147                  0.9395                  0.5338
 Frequencies --     77.4690                 81.8589                 92.3516
 IR Inten    --      0.0259                  0.2706                  0.4917
 Frequencies --    100.3549                104.2827                114.3208
 IR Inten    --      0.0488                  0.0115                  0.1629
 Frequencies --    136.4726                137.2153                137.7370
 IR Inten    --      0.0492                  0.0086                  0.0039
 Frequencies --    139.7095                140.4744                141.9106
 IR Inten    --      0.0333                  0.0005                  0.0846
 Frequencies --    155.9908                170.5154                171.9496
```

IR Inten	--	1.9922	0.0301	0.0385
Frequencies	--	175.4190	185.9492	191.1612
IR Inten	--	0.0115	0.1457	2.3789
Frequencies	--	214.9250	221.1335	221.5030
IR Inten	--	2.1940	2.3426	1.4893
Frequencies	--	226.6129	231.3775	256.9061
IR Inten	--	17.2800	0.5374	0.5532
Frequencies	--	259.9757	269.4588	273.0141
IR Inten	--	1.3237	2.6687	0.0983
Frequencies	--	273.2798	274.5280	274.9294
IR Inten	--	0.6943	0.7655	0.1325
Frequencies	--	275.1969	275.7151	276.0568
IR Inten	--	0.0781	0.5351	0.0322
Frequencies	--	276.3542	277.7292	280.3426
IR Inten	--	0.2638	0.4879	1.4941
Frequencies	--	293.4717	295.1792	301.9511
IR Inten	--	0.9900	1.3399	0.9419
Frequencies	--	303.0764	307.7489	310.2522
IR Inten	--	1.7698	0.8175	0.3176
Frequencies	--	312.6406	319.5151	323.2532
IR Inten	--	4.7865	2.9493	2.4276
Frequencies	--	326.7472	329.8603	332.1409
IR Inten	--	1.6272	1.4200	0.2218
Frequencies	--	345.9180	358.4434	373.3157
IR Inten	--	10.0811	1.5142	1.7608
Frequencies	--	376.8209	378.5628	379.2497
IR Inten	--	0.0976	0.3045	0.1851

Frequencies --	380.0238	381.8886	384.5000
IR Inten --	0.1830	0.8019	0.4189
Frequencies --	385.1513	396.9855	401.8555
IR Inten --	0.2380	0.1983	0.6295
Frequencies --	411.1622	419.2752	446.6031
IR Inten --	1.6865	7.2515	0.7592
Frequencies --	447.5097	447.7603	449.2851
IR Inten --	3.2818	1.4216	0.3084
Frequencies --	449.8262	462.3112	463.0446
IR Inten --	0.4128	1.9212	41.2895
Frequencies --	464.3254	471.1519	481.2479
IR Inten --	7.2539	0.1267	81.2261
Frequencies --	491.2834	515.6722	521.2575
IR Inten --	177.5239	15.3735	22.1769
Frequencies --	545.9449	564.4932	565.9360
IR Inten --	20.4416	27.8331	0.9505
Frequencies --	576.5669	578.6755	582.6129
IR Inten --	4.7990	0.2475	38.6693
Frequencies --	587.0906	590.9720	601.5049
IR Inten --	0.7521	0.4708	2.0099
Frequencies --	608.8259	621.1441	636.8846
IR Inten --	0.2482	25.0945	0.7353
Frequencies --	640.1284	655.0246	655.9220
IR Inten --	0.1970	0.0165	0.0313
Frequencies --	656.3213	657.7214	657.8936
IR Inten --	0.0406	0.2118	0.1771
Frequencies --	658.5557	660.5100	664.8491

IR Inten	--	0.0348	1.3060	0.0068
Frequencies	--	666.4806	673.3064	686.5146
IR Inten	--	0.1555	0.0516	0.2831
Frequencies	--	698.5510	705.3777	712.2459
IR Inten	--	2.2884	0.2279	0.8540
Frequencies	--	727.1475	752.5271	769.5502
IR Inten	--	0.2920	24.5854	1.0503
Frequencies	--	783.8306	793.6011	794.8719
IR Inten	--	0.3790	0.0987	0.3858
Frequencies	--	796.5906	800.4336	833.4745
IR Inten	--	0.0282	0.2355	0.2823
Frequencies	--	993.5115	997.2363	1001.1946
IR Inten	--	68.3160	169.4160	55.5327
Frequencies	--	1002.6739	1008.5049	1010.0170
IR Inten	--	9.4736	61.4798	30.7138
Frequencies	--	1013.0204	1013.8723	1019.3314
IR Inten	--	249.8681	574.4299	771.7358
Frequencies	--	1019.6812	1139.8667	1146.1090
IR Inten	--	10.4854	0.6505	51.0187
Frequencies	--	1150.1930	1151.3426	1156.0534
IR Inten	--	0.5715	0.6297	3.5826
Frequencies	--	1159.2298	1160.2719	1161.6407
IR Inten	--	2.3101	5.2722	8.0719
Frequencies	--	1163.4056	1165.2996	1237.2485
IR Inten	--	3.9828	2.7184	23.4700
Frequencies	--	1277.8601	1299.6511	1305.2738
IR Inten	--	766.1326	756.5674	1038.7513

Frequencies	--	1306.3923	1307.9022	1308.2828
IR Inten	--	37.7794	310.8301	5.1288
Frequencies	--	1311.2925	1312.3033	1322.1439
IR Inten	--	1043.0586	613.2755	22.2324
Frequencies	--	1322.9174	1324.7638	1327.7488
IR Inten	--	30.9704	47.8208	230.2970
Frequencies	--	1328.5068	1463.7281	1490.0037
IR Inten	--	58.4642	12.8070	9.6642
Frequencies	--	1490.2780	1502.7271	1507.0645
IR Inten	--	0.4450	478.6837	95.8222
Frequencies	--	1525.9666	1528.2571	1528.6912
IR Inten	--	45.4867	33.9592	34.5187
Frequencies	--	1531.7367	1531.8583	1533.0109
IR Inten	--	109.0418	156.5802	377.7972
Frequencies	--	1534.8674	1536.5872	1537.7491
IR Inten	--	734.7109	827.4107	755.2602
Frequencies	--	1541.5250	1550.4665	1657.1631
IR Inten	--	462.3377	147.9793	1.9807
Frequencies	--	1657.6980	1659.4900	1659.7933
IR Inten	--	2.0713	1.2869	4.6973
Frequencies	--	1665.4200	1670.4671	1670.6186
IR Inten	--	1.2723	0.8264	2.1526
Frequencies	--	1672.5077	1673.5056	1674.5882
IR Inten	--	1.4575	1.6404	0.2297

E(B3LYP) -4140.8457782 A.U.

ZPVE 0.291846 A.U.

4.2 B_5R_5, R=-C_6F_5, (^{3}A, C1)

```
C 3.331923541 -0.1234018832 2.4506622715
C 2.7882726899 0.3680314301 1.2619054619
C 3.5911111482 0.3636014661 0.1205132476
C 4.8879109117 -0.1284952618 0.1526391613
C 5.4126134037 -0.6184923588 1.342693338
C 4.6312850922 -0.6137801003 2.4931630297
O 1.525837477 0.8512174538 1.2750117424
B 0.5473844889 0.6464391165 0.3291740357
B 0.0675650139 -0.4140354123 -0.8970538281
O 0.5553970289 -1.5521302629 -1.4704400797
C 1.7238976536 -2.1458446842 -1.0962533365
C 2.7407431398 -2.3138514465 -2.0345471648
C 3.9358512612 -2.9317068805 -1.6846730569
C 4.1293257839 -3.3825838784 -0.3835037611
C 3.123034581 -3.222525084 0.5611096127
C 1.9291042002 -2.6129966764 0.2004685675
F 2.57382061 -1.8634568821 -3.277227819
F 4.9027942734 -3.0779270432 -2.5886670766
F 5.2827773458 -3.9536386024 -0.0375540604
F 3.3116066974 -3.6363770238 1.8161871478
F 0.9700265339 -2.449176491 1.1223888176
F 3.0985321492 0.8301535361 -1.0395192773
F 5.6234880916 -0.1425046334 -0.9601661243
F 6.6543379056 -1.0986352884 1.3771740382
F 5.1269213893 -1.0905521094 3.6332771744
```

F 2.5874908715 -0.1421038506 3.5554361165

B -0.1590774711 1.5687903418 -0.9371601727

O 0.2126570516 2.7787882014 -1.4431017089

C 1.2534534 3.5025459573 -0.9336189852

C 2.3791351224 3.7380265289 -1.7187308121

C 3.4501383161 4.468666889 -1.2194839558

C 3.4040777279 4.9706806302 0.076396718

C 2.2835543278 4.7467046596 0.8679726174

C 1.2155634115 4.0190673481 0.3593756603

F 2.4418486156 3.237847506 -2.9531296259

F 4.5293509121 4.6772273452 -1.9753465882

F 4.4334725501 5.6681414265 0.5590049981

F 2.2364994848 5.2290606201 2.1108367972

F 0.1395268708 3.8019058197 1.1238313844

B -1.1490923738 0.4420179576 0.3768764766

O -1.9629787163 0.3634297201 1.4683325779

C -3.1404651795 -0.2969368152 1.5887151707

C -4.194010295 0.3180815628 2.2657517387

C -5.4067328837 -0.3378098757 2.4391136587

C -5.5827241148 -1.6215809561 1.9344275722

C -4.5410563409 -2.2462426546 1.258759748

C -3.3301958415 -1.5883601518 1.0975583989

F -4.0422568354 1.5535877708 2.739145046

F -6.4063942754 0.266573604 3.0780439528

F -6.7495353536 -2.2462215346 2.0849164819

F -4.708191472 -3.471006275 0.7559559126

F -2.330483137 -2.2014157377 0.4453599917

B	-1.3702598747	0.4492186231	-1.3141943678
O	-2.3527844188	0.2771425296	-2.2611066911
C	-3.5961199657	-0.1918168999	-1.981275522
C	-3.9958198535	-1.4407179141	-2.4549725477
C	-5.2798927839	-1.915955963	-2.2139796377
C	-6.1818454214	-1.143035247	-1.4915024758
C	-5.7969362819	0.1038817543	-1.013110663
C	-4.5125797426	0.56964248	-1.2567695771
F	-3.1337059291	-2.1943758268	-3.1377702206
F	-5.6445636321	-3.1156543063	-2.6644455177
F	-7.4088981163	-1.6010908296	-1.2447732244
F	-6.6548259069	0.8408657099	-0.3032527306
F	-4.1407058566	1.7630228527	-0.7757110559

Harmonic frequencies (cm**-1), IR intensities (KM/Mole)

Frequencies	--	3.1637	9.0359	10.0649
IR Inten	--	0.0050	0.0072	0.0050
Frequencies	--	13.0105	16.1026	20.4945
IR Inten	--	0.0114	0.0306	0.0113
Frequencies	--	25.3842	26.9349	29.5481
IR Inten	--	0.0170	0.0016	0.0038
Frequencies	--	36.5138	38.9485	40.7236
IR Inten	--	0.0315	0.0116	0.0691
Frequencies	--	43.4627	54.7507	57.1533
IR Inten	--	0.0167	0.0537	0.1069
Frequencies	--	60.2195	68.8012	70.6555
IR Inten	--	0.0607	0.3075	0.0953

Frequencies	--	77.6572	86.8937	95.3861
IR Inten	--	0.0442	0.4384	0.1874
Frequencies	--	99.6620	104.5018	112.2136
IR Inten	--	0.0537	0.0115	0.0108
Frequencies	--	135.6309	137.5373	138.1141
IR Inten	--	0.0519	0.0259	0.0042
Frequencies	--	138.6934	140.2534	141.6961
IR Inten	--	0.0297	0.0083	0.0383
Frequencies	--	156.5329	168.0139	170.5930
IR Inten	--	0.4987	0.0832	0.0136
Frequencies	--	174.6839	184.6751	195.0585
IR Inten	--	0.0095	0.0507	1.3305
Frequencies	--	212.2819	216.8787	221.4249
IR Inten	--	0.4110	1.7371	9.6273
Frequencies	--	225.1492	232.1989	241.5045
IR Inten	--	9.2651	5.5084	2.5825
Frequencies	--	253.4885	265.5603	270.3375
IR Inten	--	0.8843	0.1960	0.6313
Frequencies	--	273.4138	274.3646	274.7691
IR Inten	--	0.4559	0.6489	0.3011
Frequencies	--	275.0886	275.3278	275.6872
IR Inten	--	0.2838	0.3667	0.0473
Frequencies	--	276.2216	277.5341	278.3481
IR Inten	--	0.2060	0.0392	0.1778
Frequencies	--	282.2128	291.4924	298.5458
IR Inten	--	0.6454	0.7390	0.1729
Frequencies	--	302.2005	307.2199	309.2914

IR Inten	--	1.2268	0.1664	0.9496
Frequencies	--	312.9215	315.2059	318.7500
IR Inten	--	6.3406	3.1794	1.4122
Frequencies	--	323.1370	327.6783	329.3444
IR Inten	--	4.3241	0.7054	1.5352
Frequencies	--	337.7119	351.1484	374.1390
IR Inten	--	6.4797	0.5825	0.0774
Frequencies	--	375.0653	377.8213	378.2347
IR Inten	--	0.2979	0.1779	0.1517
Frequencies	--	378.5886	381.4900	384.6890
IR Inten	--	0.0866	0.0989	0.0755
Frequencies	--	385.1140	388.4285	401.5734
IR Inten	--	0.2245	0.0760	0.5066
Frequencies	--	417.1242	428.2320	441.9405
IR Inten	--	1.1696	2.7165	1.5521
Frequencies	--	443.7253	447.5843	448.5717
IR Inten	--	1.1497	0.2048	0.3955
Frequencies	--	449.5989	452.8530	456.9755
IR Inten	--	0.4139	3.2041	1.3849
Frequencies	--	458.3410	460.4060	504.0649
IR Inten	--	2.6778	2.8563	0.1410
Frequencies	--	513.2667	529.8154	546.3056
IR Inten	--	0.7661	0.8647	1.5836
Frequencies	--	550.0404	555.6618	570.2973
IR Inten	--	4.0376	2.2746	1.1509
Frequencies	--	574.9597	578.4082	580.6954
IR Inten	--	4.0357	0.2898	1.3498

Frequencies --	585.9866	598.6544	601.6342
IR Inten --	4.6554	2.7400	4.1547
Frequencies --	615.6113	624.4418	631.3388
IR Inten --	1.8885	6.1269	7.2325
Frequencies --	644.8283	650.6517	652.8912
IR Inten --	2.6570	0.6891	0.0474
Frequencies --	653.8110	656.2153	657.2947
IR Inten --	0.0344	0.8009	0.2157
Frequencies --	657.8527	659.7134	661.8250
IR Inten --	0.2813	0.4603	3.0018
Frequencies --	665.0859	667.0980	680.6974
IR Inten --	2.3961	1.4501	2.5475
Frequencies --	684.8718	690.5739	691.8554
IR Inten --	6.4849	4.9455	92.7175
Frequencies --	704.8885	711.7912	719.0584
IR Inten --	0.4644	3.0287	0.4074
Frequencies --	738.9854	792.2037	794.1452
IR Inten --	14.1703	0.0942	0.8845
Frequencies --	794.4304	797.3374	802.2602
IR Inten --	0.0955	0.0262	1.4911
Frequencies --	988.9029	999.5633	1000.7480
IR Inten --	29.2133	106.5313	256.3819
Frequencies --	1003.1497	1003.4199	1007.3792
IR Inten --	43.5487	59.7512	18.2783
Frequencies --	1009.2639	1013.5727	1015.0748
IR Inten --	264.5281	349.8386	552.0650
Frequencies --	1020.2620	1133.6873	1147.0933

IR Inten	--	70.5054	9.1612	73.8018
Frequencies	--	1148.2450	1151.1318	1153.2492
IR Inten	--	40.2724	4.3357	1.5066
Frequencies	--	1157.0663	1158.3711	1160.9413
IR Inten	--	57.0469	7.6425	8.1737
Frequencies	--	1161.5664	1165.9715	1229.4769
IR Inten	--	2.6569	8.4817	27.8252
Frequencies	--	1254.1102	1271.9741	1282.1199
IR Inten	--	854.0379	1201.6133	2592.8987
Frequencies	--	1304.7232	1306.7811	1307.1201
IR Inten	--	6.1241	8.9199	9.1092
Frequencies	--	1308.4385	1310.1981	1322.5783
IR Inten	--	27.0553	84.0089	24.4379
Frequencies	--	1322.9180	1323.6560	1328.3264
IR Inten	--	19.6855	6.0968	94.6927
Frequencies	--	1329.3405	1456.8723	1491.8796
IR Inten	--	109.6297	4.7732	6.8812
Frequencies	--	1493.4157	1498.0264	1505.4492
IR Inten	--	7.2262	85.5382	385.7138
Frequencies	--	1521.8391	1527.7950	1527.9760
IR Inten	--	11.1847	113.7915	28.1383
Frequencies	--	1530.2169	1531.2787	1532.4253
IR Inten	--	424.9977	221.6903	50.1143
Frequencies	--	1535.1044	1535.5395	1538.0542
IR Inten	--	960.2543	281.7851	406.8629
Frequencies	--	1539.2969	1547.3692	1654.9931
IR Inten	--	986.8136	154.8180	3.7701

Frequencies --	1656.9971	1658.2479	1659.7835
IR Inten --	1.1161	3.9226	2.3076
Frequencies --	1661.9193	1669.4130	1669.8885
IR Inten --	2.1966	2.2928	4.3122
Frequencies --	1672.1293	1672.2015	1673.1309
IR Inten --	1.3573	0.8097	0.5408

E(B3LYP) -4140.8119337 A.U.

ZPVE 0.290678 A.U.

4.3 $B_5R_5{}^-$, R=-C_6F_5 (^{2}A, C1)

C	3.5684994141	-0.3875592022	2.504834868
C	2.9013926266	0.1230217215	1.3823134088
C	3.6830102966	0.3796218272	0.2481054843
C	5.0493352536	0.1357259986	0.2304387093
C	5.6833491412	-0.3706367637	1.3559874392
C	4.9347345264	-0.6309004791	2.4963564678
O	1.5950411273	0.3774695384	1.4653266989
B	0.6274835423	-0.0433752235	0.5026346283
B	0.3470669014	-0.9990638512	-0.8040658186
O	1.0602902593	-1.9678210529	-1.5546588453
C	2.3210579958	-2.3079983429	-1.2451952785
C	3.3386873708	-2.1370183073	-2.1894787157
C	4.6477540296	-2.5181121974	-1.9259008682
C	4.9748771606	-3.0759266684	-0.6971979675
C	3.9829353672	-3.2563297847	0.2563928867
C	2.6747084114	-2.8812864092	-0.0187489267

F	3.0532231369	-1.5874087623	-3.3751427127
F	5.6016575051	-2.338061197	-2.8492657813
F	6.2399808633	-3.4317590578	-0.4306320603
F	4.2939896021	-3.7908520723	1.4464417571
F	1.7386995209	-3.0849773707	0.9173477955
F	3.1146552237	0.8835715683	-0.8597364856
F	5.7597429436	0.3882841061	-0.8776953695
F	7.0027926594	-0.6075610958	1.3415579711
F	5.5362845814	-1.1267776465	3.5861870083
F	2.8727904527	-0.6599333197	3.6146402825
B	-0.2527609343	0.7848613797	-0.7154140988
O	-0.1335609049	2.0625628073	-1.2583631836
C	0.7440100153	2.9555707953	-0.7464642802
C	1.7819227558	3.4438499321	-1.5406831937
C	2.697465389	4.3610998736	-1.0435846672
C	2.5899086127	4.8084995394	0.2665123202
C	1.5569709384	4.3433532649	1.0692890283
C	0.6409460725	3.430424371	0.5630349494
F	1.9170095615	3.0069563319	-2.7966321071
F	3.6957208342	4.8073922362	-1.8193971131
F	3.4736804488	5.6940485349	0.7507241637
F	1.4423016482	4.7870226073	2.3282398857
F	-0.3574754713	3.011185335	1.3442241595
B	-1.0494243179	-0.2215802144	0.5906812708
O	-1.8540072315	-0.1615161205	1.7491770031
C	-3.1849328165	-0.3385444885	1.7012251826
C	-4.0382361013	0.6981932	2.0913540929

C -5.41763162 0.5374276948 2.1018158397

C -5.9791643485 -0.6707123828 1.7107183176

C -5.1522635285 -1.7141328172 1.3188669109

C -3.7738736214 -1.5476996492 1.3187703705

F -3.5223444974 1.8753829109 2.459711177

F -6.2119204031 1.5486421945 2.4779963981

F -7.3106520408 -0.827179981 1.7051437816

F -5.6900059983 -2.8808269084 0.9351104243

F -3.0012160016 -2.5754336581 0.9453270205

B -1.2608660456 -0.5435185818 -1.0252352717

O -2.245546238 -0.9008176441 -1.9917279735

C -3.5552784598 -0.6985708908 -1.8607509215

C -4.4368285659 -1.7296998555 -2.2182503903

C -5.8137254853 -1.5760107088 -2.1452161487

C -6.3592778522 -0.3779862317 -1.7033099139

C -5.5111379906 0.6593438583 -1.3432764043

C -4.1339704645 0.5029054859 -1.4270723001

F -3.9418206564 -2.9019137369 -2.6337002788

F -6.6231951808 -2.5874658291 -2.4899033634

F -7.6896843 -0.2250966234 -1.6247160838

F -6.0266545541 1.820474112 -0.9132174424

F -3.3563215592 1.5430979017 -1.0927167366

Harmonic frequencies (cm**-1), IR intensities (KM/Mole)

Frequencies --	9.3244	11.5593	12.6055
IR Inten --	0.0233	0.0060	0.0055
Frequencies --	14.9287	18.7557	20.4031

IR Inten	--	0.0184	0.0365	0.0144
Frequencies	--	26.9651	29.8364	31.8997
IR Inten	--	0.1526	0.1780	0.2270
Frequencies	--	36.1637	39.0064	42.3856
IR Inten	--	0.1113	0.0539	0.0837
Frequencies	--	43.5827	57.3688	59.3574
IR Inten	--	0.0258	0.0928	0.0359
Frequencies	--	61.9324	67.9120	73.6380
IR Inten	--	0.7745	0.1326	0.1438
Frequencies	--	79.4560	85.7483	95.1232
IR Inten	--	0.0856	0.2271	0.1134
Frequencies	--	100.2293	104.2748	113.2907
IR Inten	--	0.0477	0.0426	0.0723
Frequencies	--	136.5610	137.1472	138.1219
IR Inten	--	0.0248	0.0112	0.0445
Frequencies	--	139.0362	140.5288	142.3951
IR Inten	--	0.0568	0.0044	0.0093
Frequencies	--	156.9660	171.5596	172.3088
IR Inten	--	0.8015	0.1093	0.0071
Frequencies	--	176.2138	184.8996	194.6684
IR Inten	--	0.0131	0.0296	1.3597
Frequencies	--	213.9399	218.0218	222.7621
IR Inten	--	3.2140	1.4130	5.8144
Frequencies	--	228.1975	230.9750	247.1641
IR Inten	--	5.2827	3.7893	2.7509
Frequencies	--	253.3870	266.2960	273.1955
IR Inten	--	1.2487	1.8507	0.8806

Frequencies	--	274.5705	274.9234	275.1524
IR Inten	--	0.3405	0.2616	0.0866
Frequencies	--	275.4902	275.6080	275.9721
IR Inten	--	0.3096	0.4221	0.0476
Frequencies	--	278.2108	278.5851	279.0579
IR Inten	--	0.5350	0.3332	0.1614
Frequencies	--	286.8602	294.1735	300.8471
IR Inten	--	1.3008	1.4752	0.7990
Frequencies	--	302.2545	306.4021	311.6617
IR Inten	--	2.1468	0.0484	2.4624
Frequencies	--	312.9355	315.0138	321.9093
IR Inten	--	3.7828	3.3244	3.1615
Frequencies	--	323.7413	327.9156	330.7107
IR Inten	--	1.6921	0.5689	0.0433
Frequencies	--	344.2397	355.6189	370.6308
IR Inten	--	1.3214	0.7374	0.1971
Frequencies	--	372.3603	375.4929	377.0063
IR Inten	--	0.8565	0.4236	0.5902
Frequencies	--	377.7964	381.2711	382.2067
IR Inten	--	0.2225	1.9241	0.3333
Frequencies	--	382.9621	392.7095	405.2792
IR Inten	--	0.5246	0.4923	2.5626
Frequencies	--	428.5287	429.1155	444.9575
IR Inten	--	10.1970	0.2113	2.0495
Frequencies	--	445.9316	447.1801	448.6697
IR Inten	--	0.8987	4.3934	0.0878
Frequencies	--	450.6455	457.2099	460.0128

IR Inten	--	0.5716	10.8310	1.1747
Frequencies	--	462.9424	466.4781	477.1873
IR Inten	--	1.8539	0.4673	47.9455
Frequencies	--	499.0333	508.6010	523.6645
IR Inten	--	55.2731	14.0106	2.4735
Frequencies	--	539.8228	558.9702	565.9697
IR Inten	--	11.0463	10.1776	4.0564
Frequencies	--	573.8134	575.4386	576.3582
IR Inten	--	9.7555	2.5156	3.3364
Frequencies	--	584.7751	594.6236	599.2239
IR Inten	--	1.3634	1.4362	0.8569
Frequencies	--	610.8183	621.4800	639.9953
IR Inten	--	6.0316	3.3956	0.7453
Frequencies	--	643.6532	649.2995	649.9306
IR Inten	--	0.5486	0.2432	0.0251
Frequencies	--	654.2213	655.3649	656.1566
IR Inten	--	0.3708	0.2207	0.0930
Frequencies	--	659.6301	661.7588	663.7353
IR Inten	--	0.0631	1.1121	1.0476
Frequencies	--	665.1082	671.4649	678.4605
IR Inten	--	1.2144	0.2053	2.2491
Frequencies	--	698.9599	713.7644	719.6524
IR Inten	--	1.3043	1.5020	1.8802
Frequencies	--	727.1281	735.5795	745.0891
IR Inten	--	0.7973	0.0899	2.1124
Frequencies	--	774.7581	794.0028	795.6552
IR Inten	--	2.6632	0.2432	0.1468

Frequencies	--	797.1240	801.3669	830.5007
IR Inten	--	0.1729	0.1658	2.6127
Frequencies	--	985.4514	995.6273	996.3479
IR Inten	--	44.0123	77.8535	13.4122
Frequencies	--	999.0292	1003.0140	1007.7764
IR Inten	--	163.6266	55.3319	383.8778
Frequencies	--	1008.4385	1008.9279	1019.4253
IR Inten	--	260.9182	296.1692	285.7984
Frequencies	--	1019.7629	1087.3626	1137.2731
IR Inten	--	655.9239	9.9272	93.9437
Frequencies	--	1137.9414	1140.2907	1145.1627
IR Inten	--	1.1578	184.3890	1.6859
Frequencies	--	1146.5683	1149.1970	1155.9116
IR Inten	--	0.9078	3.3713	18.1139
Frequencies	--	1166.2816	1169.2346	1176.6527
IR Inten	--	22.7981	5.7653	26.6408
Frequencies	--	1191.1223	1202.3918	1256.3399
IR Inten	--	708.9491	675.3907	2140.1992
Frequencies	--	1302.9222	1303.5218	1304.7863
IR Inten	--	12.1724	27.2760	1.1308
Frequencies	--	1304.8798	1306.3164	1316.9455
IR Inten	--	8.3117	4.6645	12.6723
Frequencies	--	1318.1992	1319.0812	1321.9278
IR Inten	--	19.7399	3.6002	3.7988
Frequencies	--	1322.8192	1399.8294	1483.7682
IR Inten	--	105.9492	5.4137	8.4026
Frequencies	--	1484.3754	1490.3946	1499.2540

IR Inten	--	6.3662	62.6738	430.1439
Frequencies	--	1505.9607	1521.9821	1522.1824
IR Inten	--	18.2353	131.6246	82.4683
Frequencies	--	1522.9003	1523.1962	1525.1978
IR Inten	--	47.2295	38.1266	369.7508
Frequencies	--	1526.9877	1528.2548	1528.8891
IR Inten	--	177.1858	359.2719	1808.2307
Frequencies	--	1531.9949	1539.4136	1645.9805
IR Inten	--	764.9219	11.5351	4.8600
Frequencies	--	1646.7602	1651.6316	1652.7556
IR Inten	--	5.6347	3.8916	3.8102
Frequencies	--	1657.2047	1668.3415	1668.5138
IR Inten	--	2.1570	8.2241	5.9799
Frequencies	--	1670.7869	1671.2587	1672.6256
IR Inten	--	25.3435	3.3943	0.3220

E(B3LYP) -4140.9640973 A.U.

ZPVE 0.289830 A.U.

4.4 $B_5R_5^-$, R=-C_6F_5 (^{4}A, C1)

C	2.075932045	-1.3019795519	2.4356876829
C	2.3502535651	-0.084866639	1.8029229834
C	3.6696865658	0.1543799484	1.4088934325
C	4.6777359082	-0.7702048071	1.6425889611
C	4.3838565695	-1.9704096806	2.2754280108
C	3.0786273934	-2.2347521936	2.669545779
O	1.4105915983	0.8506236794	1.5875972056

B	0.3413274179	0.5867136498	0.6960293226
B	0.3177461868	0.1325994988	-0.9565185135
O	1.3410889192	-0.0769841464	-1.9092971512
C	2.4067594003	-0.8518588136	-1.6782520754
C	3.6661617557	-0.4058054398	-2.0996664352
C	4.8117866542	-1.1657923487	-1.9108222274
C	4.7301224345	-2.402197439	-1.2845663311
C	3.4940681432	-2.8662883672	-0.8563018497
C	2.3489794634	-2.1060994723	-1.0545250524
F	3.7756580194	0.7902977377	-2.6900827719
F	6.0001095769	-0.703775391	-2.3203050422
F	5.8329795565	-3.1398678591	-1.0933741321
F	3.4069225854	-4.0579112968	-0.2481332751
F	1.175708061	-2.6051399124	-0.6497279137
F	3.9699354321	1.2915765444	0.765045446
F	5.9304636445	-0.5175275404	1.239282846
F	5.3525314401	-2.8680589084	2.501452226
F	2.7928067188	-3.3884744866	3.2859678481
F	0.8324601926	-1.5727531756	2.8419763577
B	-0.3833231935	1.7004284127	-0.4816283968
O	-0.124613427	3.0480459668	-0.86097424
C	1.170065347	3.4287840965	-0.9001290872
C	1.8915526225	3.4032495236	-2.0964010406
C	3.2200264678	3.8037863822	-2.1480509533
C	3.8596494728	4.2336348539	-0.9937878488
C	3.160918927	4.272856848	0.2048322871
C	1.8315496976	3.8773070761	0.2464350046

F	1.2958777727	2.9871270461	-3.2195067167
F	3.8899511763	3.7784493044	-3.308834389
F	5.1453195031	4.6166208185	-1.0375612813
F	3.7768865262	4.6921543729	1.3208438591
F	1.1754311122	3.9308289364	1.4112078532
B	-1.355841886	0.792318531	0.6759153155
O	-2.3528381687	1.2069109117	1.584511144
C	-3.5103611259	0.5486695752	1.7232175906
C	-4.6982148541	1.2852795988	1.8181259053
C	-5.929642654	0.6659781695	1.9815569791
C	-6.0095204581	-0.7182441169	2.0507053647
C	-4.84666011	-1.4704907203	1.960976046
C	-3.6156742237	-0.8471586953	1.8038574548
F	-4.653057093	2.6199666342	1.7396746677
F	-7.0468319916	1.4018119445	2.060309578
F	-7.1964997795	-1.3226039347	2.2030237064
F	-4.9143654393	-2.8079229683	2.0278705429
F	-2.518908247	-1.6109651996	1.7505012962
B	-1.3807263174	0.3468119629	-0.9844579808
O	-2.4223560833	0.2335673433	-1.9337664426
C	-3.4511221872	-0.5961748458	-1.6855030337
C	-3.298748116	-1.9839336356	-1.5909436923
C	-4.3873908013	-2.8151942916	-1.3579895613
C	-5.6591959661	-2.2763768978	-1.2155336096
C	-5.8328328186	-0.902004972	-1.3048156173
C	-4.7402059919	-0.0768909098	-1.5333107337
F	-2.0901928951	-2.5318427191	-1.7448152421

F	-4.2161516387	-4.1409073196	-1.2804252707
F	-6.7109923373	-3.0759384042	-0.992088596
F	-7.054842307	-0.3729248549	-1.1479908454
F	-4.9302187617	1.2474665873	-1.5867583469

Harmonic frequencies (cm**-1), IR intensities (KM/Mole)

Frequencies --	3.3659	7.6130	11.3491
IR Inten --	0.0308	0.0208	0.0284
Frequencies --	12.2342	20.8826	25.3771
IR Inten --	0.0195	0.0006	0.0207
Frequencies --	27.8761	29.2626	29.8215
IR Inten --	0.0019	0.0450	0.0313
Frequencies --	30.7161	36.5806	38.1128
IR Inten --	0.0570	0.1401	0.0310
Frequencies --	41.4374	55.8381	56.3981
IR Inten --	0.0584	0.1222	0.0339
Frequencies --	62.3539	67.8051	71.4741
IR Inten --	0.1973	0.1713	0.0123
Frequencies --	77.4012	81.4028	88.8080
IR Inten --	0.0072	0.1175	0.0849
Frequencies --	97.8166	101.0418	109.7867
IR Inten --	0.0042	0.0073	0.0045
Frequencies --	131.5477	136.9312	137.4624
IR Inten --	0.0038	0.0066	0.0047
Frequencies --	138.6166	139.5881	140.2735
IR Inten --	0.0017	0.0183	0.0058
Frequencies --	166.1169	166.5994	172.1420

IR Inten	--	0.2419	0.0971	0.0086
Frequencies	--	174.8437	183.1629	202.0798
IR Inten	--	0.0019	0.0206	0.5511
Frequencies	--	206.1582	213.7553	220.4161
IR Inten	--	0.5466	0.6268	18.2552
Frequencies	--	225.8641	234.4445	237.7267
IR Inten	--	0.6893	2.8438	2.3054
Frequencies	--	244.7654	269.8907	272.6362
IR Inten	--	2.7959	0.1592	0.1814
Frequencies	--	273.4101	274.3864	274.8004
IR Inten	--	0.2720	0.0261	0.0146
Frequencies	--	274.8983	275.8615	276.3330
IR Inten	--	0.0723	0.6672	0.0561
Frequencies	--	277.4503	278.9426	282.1886
IR Inten	--	1.2193	0.0119	0.3844
Frequencies	--	284.8689	286.8488	294.3789
IR Inten	--	0.2631	0.0496	0.1759
Frequencies	--	302.8002	306.2654	309.8607
IR Inten	--	0.1115	2.6834	0.3708
Frequencies	--	312.0930	315.2217	320.8490
IR Inten	--	0.6381	6.7942	1.5472
Frequencies	--	324.0864	324.3296	326.0739
IR Inten	--	4.4521	2.3838	0.3273
Frequencies	--	328.6834	352.2931	368.9132
IR Inten	--	0.8069	0.1037	0.0665
Frequencies	--	369.5547	374.0121	375.1127
IR Inten	--	0.1019	1.4437	0.8001

Frequencies --	377.9726	378.5497	378.7860
IR Inten --	0.3427	0.0212	0.0188
Frequencies --	380.2876	381.9499	388.5374
IR Inten --	0.0446	0.0410	0.0804
Frequencies --	413.8490	427.3246	441.1388
IR Inten --	0.7130	4.0814	2.3067
Frequencies --	446.9100	447.8372	448.5968
IR Inten --	1.6211	0.0650	0.6364
Frequencies --	449.2359	450.4036	456.0594
IR Inten --	0.2605	0.8952	1.5433
Frequencies --	462.1796	475.7322	483.4061
IR Inten --	0.1744	0.0617	0.0393
Frequencies --	490.6502	499.4024	530.9393
IR Inten --	0.3573	10.1046	0.8077
Frequencies --	546.4421	550.3299	558.9702
IR Inten --	13.7888	3.3259	13.9058
Frequencies --	570.0881	574.2643	575.3666
IR Inten --	11.9088	2.1231	1.2795
Frequencies --	582.3661	583.9727	596.0076
IR Inten --	5.2773	6.1007	6.2890
Frequencies --	606.0915	614.9925	631.8461
IR Inten --	3.7197	0.0699	3.7944
Frequencies --	639.1560	649.7425	650.6798
IR Inten --	0.6137	1.4873	6.5578
Frequencies --	651.1885	652.5238	653.4739
IR Inten --	0.2944	17.6993	5.8509
Frequencies --	655.2409	657.1621	659.5987

IR Inten	--	36.6572	6.8810	0.6156
Frequencies	--	662.9755	666.4917	677.1683
IR Inten	--	4.7679	10.1561	1.8226
Frequencies	--	679.3075	704.5617	707.3290
IR Inten	--	5.7896	0.0593	1.5935
Frequencies	--	717.8749	725.7948	742.4378
IR Inten	--	0.5748	4.4220	2.4021
Frequencies	--	787.6719	790.9865	792.2400
IR Inten	--	0.0751	0.2055	0.3347
Frequencies	--	797.0521	799.6107	848.0780
IR Inten	--	0.0392	0.8637	0.4460
Frequencies	--	987.2170	994.5480	995.9321
IR Inten	--	15.4570	93.5080	250.8908
Frequencies	--	997.8216	998.9414	999.6749
IR Inten	--	51.5206	69.5193	106.8690
Frequencies	--	1002.9150	1007.6671	1009.6850
IR Inten	--	64.9443	70.8204	573.6413
Frequencies	--	1018.2835	1092.4137	1111.9473
IR Inten	--	199.3015	34.4288	2089.9018
Frequencies	--	1126.1169	1132.5290	1139.8954
IR Inten	--	354.1081	216.2106	2.4823
Frequencies	--	1141.0661	1144.2697	1146.8802
IR Inten	--	6.0034	4.5609	9.7913
Frequencies	--	1148.6616	1162.9653	1171.8450
IR Inten	--	16.5956	86.8145	201.3217
Frequencies	--	1178.0088	1180.1653	1192.0184
IR Inten	--	127.0777	714.1944	917.9164

Frequencies --	1300.6310	1301.8129	1302.3757
IR Inten --	3.8269	7.1125	15.1165
Frequencies --	1304.8917	1305.6397	1314.1968
IR Inten --	5.1293	9.2868	20.7647
Frequencies --	1317.7772	1319.1319	1321.0130
IR Inten --	0.4511	31.1550	2.5702
Frequencies --	1321.3256	1363.2083	1486.1788
IR Inten --	96.6166	14.7831	45.2428
Frequencies --	1486.8027	1489.1278	1500.4805
IR Inten --	4.4953	4.5766	286.8940
Frequencies --	1505.1794	1521.4575	1522.6495
IR Inten --	7.6895	9.8358	82.1292
Frequencies --	1523.3116	1524.1302	1524.3427
IR Inten --	31.3396	38.7400	197.1511
Frequencies --	1526.7556	1527.2731	1529.1396
IR Inten --	175.7934	646.7987	1250.2106
Frequencies --	1530.1712	1540.1873	1647.8283
IR Inten --	1032.0689	424.4671	3.6534
Frequencies --	1648.2304	1651.2500	1651.4928
IR Inten --	3.0259	6.6265	5.1228
Frequencies --	1653.4951	1667.2216	1667.7953
IR Inten --	4.0413	1.6258	9.8677
Frequencies --	1669.2503	1670.4080	1671.9496
IR Inten --	33.2443	3.1067	0.2474

E(B3LYP) -4140.8826387 A.U.

ZPVE 0.288468 A.U.
